# Lanthanide Oxalates: From Single Crystals to 2D Functional Honeycomb Nanosheets

**DOI:** 10.1021/acs.inorgchem.4c04293

**Published:** 2025-02-18

**Authors:** Daniela Veronika Uríková, Giannis Kampitakis, Ivana Císařová, Adam Alemayehu, Matouš Kloda, Dominika Zákutná, Kamil Lang, Jan Demel, Václav Tyrpekl

**Affiliations:** † Department of Inorganic Chemistry, Faculty of Science, Charles University, Prague, Hlavova 2030, Czech Republic; ‡ Institute of Inorganic Chemistry of the Czech Academy of Sciences, Husinec-Řež 1001 25068, Czech Republic

## Abstract

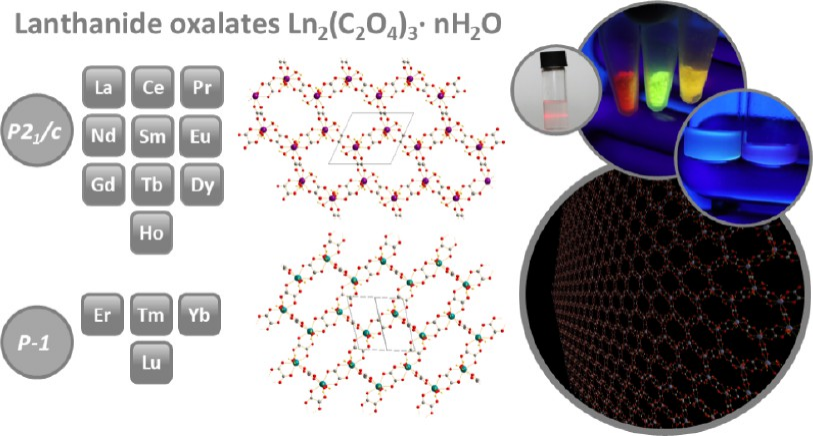

Oxalates are simple, low-cost but crucial compounds in the technology of lanthanides, actinides, and transition metals. Apart from using oxalate as a versatile ligand in coordination chemistry, simple oxalate salts are still under a scientific focus, linked with ion batteries, optical and magnetic materials, and, most importantly, industrial-technological mining and separation loops. The typically low solubility of oxalate salts is advantageous from the viewpoint of a convenient and affordable synthesis requiring only green solvents. Even though basic lanthanide oxalates have been known for decades, their structural descriptions have remained fuzzy, especially concerning water content and heavy lanthanide analogues. Herein, we present a newly developed preparation technique for large oxalate monocrystals applied to the whole lanthanide series. All of the structures were reviewed, and some new structures were determined. All of these oxalates exhibit a honeycomb structure with closed cavities containing water molecules. These honeycomb coordination polymers form a layered structure bonded by hydrogen bonds. Surprisingly, most oxalates can be easily exfoliated/delaminated in EtOH, forming colloids of up to single-layered nanosheets. Such a feature has never been described for 2D lanthanide oxalates and demonstrates a new form of applicability for them, e.g., for the construction of thin films or inkjet-printed layers using an extremely facile and economical preparation route.

## Abstract 2

The study summarizes a novel method for synthesizing large monocrystals of lanthanide oxalates and reveals their honey comb-like structure with water-filled cavities. These oxalates exhibit unexpected exfoliation in ethanol and hence form nanosheets.

## 1 Introduction

Lanthanides (Ln)^1^ represent a crucial part of modern technology in our daily life, namely in digital technology as phosphors, lamps, lasers, up-converting materials, or magnets.^2−5^ Additionally, lanthanide-based materials find applications in petroleum chemistry, catalysis^6−8^ and biomedicine.^9^ Regarding coordination compounds, lanthanide ions generally have a higher charge-to-volume ratio than d-transition metals, resulting in a preference for higher coordination numbers (8–12). Down in the series, the preference for a decreasing coordination number is observed as the Ln ions become smaller, hence harder to access by the ligands.^1^ Therefore, their rich coordination chemistry is often utilized in compound design and development, lately projected into the chemistry of porous coordination polymers, known as metal–organic frameworks (MOFs).^10−12^

Generally, lighter lanthanides are extracted from monazites, heavy lanthanides from xenotime minerals, while from euxenite all lanthanides can be extracted.^13^ There are many methods for lanthanide isolation and their variations, among which industries choose according to the ore composition, distribution of the individual Ln in the concentrate, or availability of resources. Many of the processes, e.g., the Baotou,^14^ Molycorp bastnaesite, Rhone-Poulenc, liquid–liquid extraction, or Mintek-apatite processes,^15^ employ oxalates as precipitating agents once the raw feedstocks have been digested by inorganic aqueous acids. The oxalic precipitation is not just efficient but also simple, inexpensive, and water based. It can be used during both extractions^16^ or separations.^17^ All this is due to the low solubility of oxalate salts, Ln_2_(C_2_O_4_)_3_·*n*H_2_O. For example, the solubility product of Nd(III) oxalate equals 3 × 10^–27^,^18^ which leads to concentrations <10^–3^ mol·L^–1^ in saturated solutions. Not only are oxalates of lanthanides in the R&D and industry spotlight, but also those of actinides^19^ and other transition metals.^20,21^ Additionally, owing to their central metal, lanthanide oxalates have broad application potential in advanced functional materials, e.g., organic light-emitting diodes,^22^ nonlinear optics,^23,24^ molecular sorption,^25^ or single molecular magnets.^26,27^

In many applications, thin film preparation is part of device fabrication. Thin films are often prepared by the sol–gel technique or various physical deposition techniques, such as chemical vapor deposition, laser deposition technique, etc. In recent years, exfoliation of 2D materials has proved to be a convenient way to prepare colloids containing single-layered nanosheets, which can be conveniently used for not only thin films but also functional devices.^28−30^ A prominent example of these materials is graphene;^31^ however, the group of 2D materials that can be exfoliated is growing fast, including MoS_2_, WS_2_, MoSe_2_, MoTe_2_, TaSe_2_, NbSe_2_, NiTe_2_, BN, Bi_2_Te_3_, and layered hydroxides.^32−35^ Recently, exfoliation of coordination polymers was attempted by several groups; however, a wide distribution of sheet thicknesses was commonly obtained. Interestingly, to the best of our knowledge, lanthanide oxalates have not been exfoliated, even though they represent a cheap and highly functional group of coordination polymers.

The oxalate ligand can be a bridging coligand in various 2D, 3D, and other structures. However, the present work is focused on “simple” lanthanide oxalates Ln_2_(C_2_O_4_)_3_·*n*H_2_O, where the term simple means the elementary oxalate salt of the Ln(III) ion and water. Water can be part of the primary coordination sphere or exist as a free molecule in the crystal lattice. Lanthanide oxalates are typically prepared under ambient conditions, not hydrothermally. These compounds, often considered primitive and mastered, have been studied for over 100 years; however, precise structures and water content of some of them have not been reported until now. Early works by Wyrouboff declared that La, Ce, and Nd oxalates are 11-hydrates.^36,37^ Shortly after, Wirth claimed them to be rather a 9-hydrate (Ce) and a 14-hydrate (Er).^38^ Later, in the mid-20th century, Gilphin and McCrone revealed the crystal structure of La oxalate as a 10-hydrate,^39^ nevertheless, the structure determination was somewhat inconsistent. In the 1960s–70s, it was agreed that there are two isomorphous series of Ln oxalates obtained under ambient precipitation conditions. The light elements were identified as isomorphous decahydrates, and for the heavy lanthanides, there is a second isomorphous series—hexahydrates. The transition between the two is somewhere between Ho and Er and is not sharp—as the crystallization of Dy, Ho, and Er could result in the structure of both forms.^40^ In parallel, Ollendorff studied oxalates from La to Dy, showing similar structures^41^ (data summarized in Tables S1 and S2). Still, most of the knowledge about this topic is based on powder X-ray diffraction, since studies using single crystal X-ray require high-quality single crystals of a minimal size of several tens of micrometers, which, except for La, proved to be very difficult to produce. This is because most of these salts, prepared from their aqueous solutions, are formed briskly and can be obtained only as microcrystalline powders.^42^ Up to now, there have been some advances in the field of simple oxalate structures; notably, Huang^43^ deciphered precisely the structure of La oxalate decahydrate, and Shu-Feng Si^44^ of Tm oxalate hexahydrate. However, the reported total hydration numbers ranged from 2 to 18, depending on the synthesis, drying technique, storage, and analytical tool chosen for the measurement (for more details, see Supporting Information, Discussion on the water content). A comparable situation prevails in the area of actinide oxalates, as indicated by new studies on corresponding tetravalent ions.^45^

In the present study, we employed our recently developed synthetic route for the preparation of large oxalate crystals based on homogeneous precipitation induced by acid-catalyzed oxamic acid hydrolysis^46^ to prepare the whole series of lanthanide oxalates. The resulting crystals were suitable for structure determination by single-crystal X-ray diffraction. Additionally, we demonstrate the ability of lanthanide oxalates to be exfoliated up to single-layered nanosheets, together with a brief example of their application as luminescent and magnetic materials.

## 2 Experimental Section

### 2.1 Synthesis of Hydrated La Oxalate Crystals

#### 2.1.1 Synthesis Methodology

All reagents were commercially purchased and used without further purification (for details see ref. (46)). For the synthesis, La(NO_3_)_3_·6H_2_O (Thermo Scientific, 99.9%), Ce(NO_3_)_3_·6H_2_O (Alfa Aesar, 99.9%), Pr(NO_3_)_3_·6H_2_O (Sigma-Aldrich, 99.9%), Nd(NO_3_)_3_·6H_2_O (Sigma-Aldrich, 99.9%), Sm_2_(SO_4_)_3_·8H_2_O (Alfa Aesar, 99.9%), Eu(NO_3_)_3_·6H_2_O (Strem Chemicals, 99.9%), Gd(NO_3_)_3_·6H_2_O (Alfa Aesar, 99.99%), Tb(NO_3_)_3_·6H_2_O (Sigma-Aldrich, 99.9%), Dy(NO_3_)_3_·5H_2_O (Alfa Aesar, 99.9%), Ho(NO_3_)_3_·5H_2_O (REacton, 99.9%), Er(NO_3_)_3_·5H_2_O (Sigma-Aldrich, 99.9%), Tm(NO_3_)_3_·5H_2_O (Sigma-Aldrich, 99.9%), Yb(NO_3_)_3_·5H_2_O (Sigma-Aldrich, 99.9%), Lu(NO_3_)_3_·*n*H_2_O (Sigma-Aldrich, 99.9%), oxamic acid (Alfa Aesar, 98%), 96% ethanol, and nitric acid (both Lach:ner, 65%/w) were used.

In an Eppendorf tube, 1.1 mL of a 0.5 M solution (0.55 mmol) of lanthanide nitrate in 0.01 M nitric acid was mixed with 1.55 mol equivalents of oxamic acid (75 mg, 0.85 mmol) and carefully heated to 40 °C to increase the dissolution of oxamic acid. After the oxamic acid was dissolved, yielding a transparent solution, the temperature was raised to 85 °C. The reaction mixture was kept at this temperature for around 7 h. A colorless/colored (Pr–green, Nd–violet, Sm–yellowish, Er, Ho–pink) precipitate was formed. The supernatant was removed, and the crystalline product was washed with distilled water and centrifuged for 5 min at 5000 rpm twice. The precipitate was left to dry at room temperature overnight.

In a typical exfoliation experiment, 5 mg of lanthanide oxalate was topped up with 3 mL of ethanol (96% purity) in an Eppendorf tube and kept in a laboratory ultrasound bath for 5 min. For atomic force microscopy investigations, a dispersion of 1 mg of oxalate in 1 L of ethanol was made and sonicated in a laboratory ultrasound bath for 2 h.

### 2.2 Instrumental Methods

Optical micrographs were recorded on a Leica DM4000 microscope equipped with a Leica DFC295 camera. The morphology of the produced Ln oxalates was studied under a scanning electron microscope, JEOL JSM-6510. Thermogravimetric analyses (TGA) were carried out using a SETSYS Evolution 1750 thermal analyzer under an air atmosphere (15N_2_, 5O_2_). They were performed in a 100 μL alumina crucible in the range of 20–1000 °C with a heating rate of 10 °C/min. The carrier gas used was N_2_. The thermogravimetric measurements were performed for all the synthesized samples (see Supporting Information). Diffuse reflectance spectra were measured from 300 to 600 nm on a PerkinElmer Lambda750 spectrometer equipped with a 10 cm integration sphere. Recorded reflectance values were transformed into Kubelka–Munk units. Luminescence properties were monitored on a Fluorolog 3 spectrometer equipped with a cooled TBX-05-C photon detection module (Horiba Jobin Yvon). Luminescence quantum yields (F_L_) of powders and deposited nanosheets were recorded using a Quantaurus QY C11347–1 spectrometer (Hamamatsu, Japan). The emission bands were measured in the visible region (550–750 nm for Eu oxalate and 450–750 nm for Tb oxalate) using excitation wavelengths of 395 and 370 nm for Eu oxalate and Tb oxalates, respectively.

Powder X-ray diffraction data were collected on a PANalytical X’Pert PRO diffractometer for Cu Kα radiation (λ = 1.5406 Å) in the standard Bragg–Brentano setup, calibrated on a LaB_6_ (NIST) standard. The pattern was treated by using Jana 2006 software. Structural characterization was attained using a single crystal diffractometer D8 Venture Kappa Duo (Bruker) with a Mo anode and a monochromatizing mirror selecting the wavelength λ = 0.71073 Å. The received data were treated using Bruker Apex and Bruker SAINT software, subsequently solved by the SHELXT 2014/6 program, and refined by implementing the least-squares method of SHELXL 2017 software.

Atomic force microscopy (AFM) was performed on a Dimension Icon microscope (Bruker, USA) in ScanAsyst mode using ScanAsyst air cantilevers. For AFM measurement, 80 μL of the delaminated oxalate dispersion was placed on the freshly cleaved mica surface (AFM sample holder) using the spin coating method. Measurements were done for the oxalates of Eu, Tb, Er, Tm, Yb, and Lu.

Magnetization measurements were performed using a Physical Property Measurement System (PPMS) from Quantum Design (QD) equipped with a Vibrating Sample Magnetometry (VSM) module. The samples were measured inside a plastic sample holder while being glued to avoid the movement of the crystallites. The magnetization change with temperature was recorded in the temperature range of 2–300 K under zero-field cooled (ZFC) and field-cooled (FC) conditions with an applied field of 100 mT (and 1 T for the Tb sample). The magnetization vs applied field curves were registered at a temperature of 4 K, in the range of the applied magnetic field of ±8 T. The Tb sample was measured on a different instrument of the same type.

Additional information about the software used is found in the Supporting Information.

## 3 Results and Discussion

### 3.1 Ln_2_(C_2_O_4_)_3_·*n*H_2_O Nature and Structure

**Figure 1:**
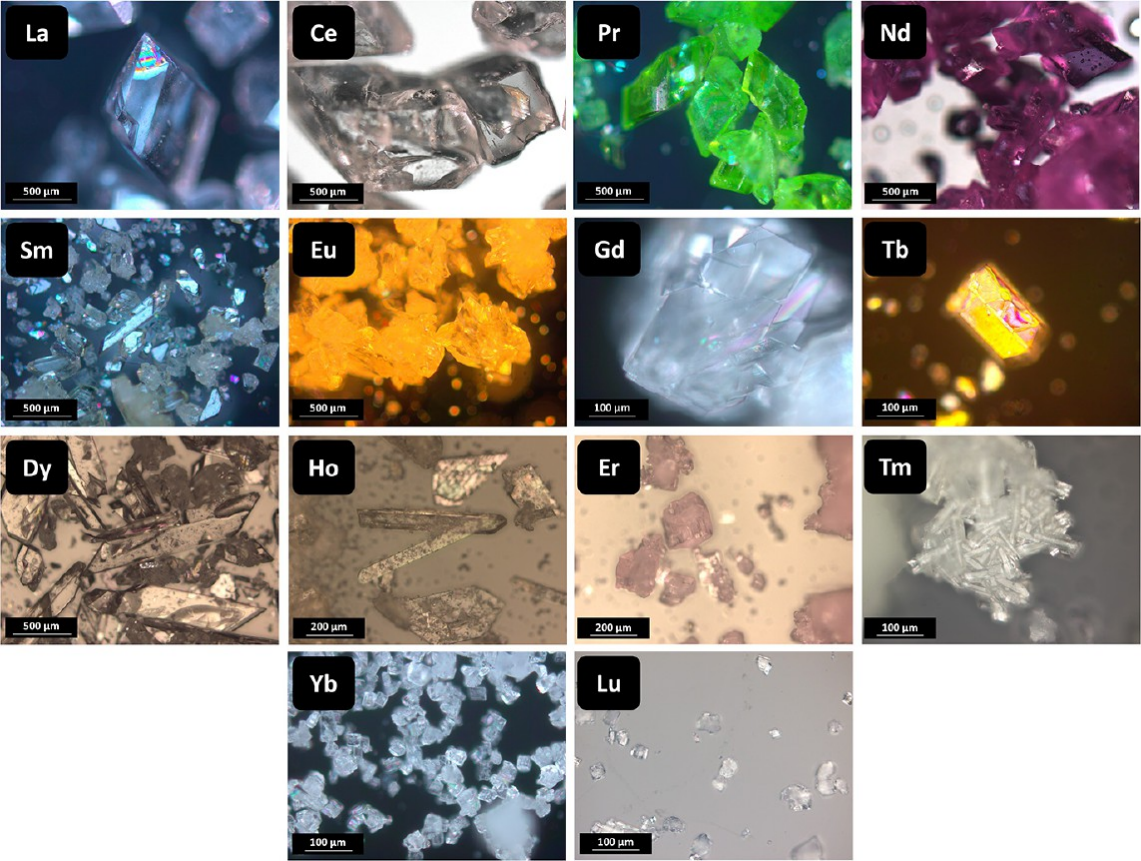
Optical micrographs of large crystals of Ln_2_(C_2_O_4_)_3_·*n*H_2_O prepared by homogeneous precipitation.

Large Ln_2_(C_2_O_4_)_3_·*n*H_2_O (Ln from lanthanum to lutetium, except radioactive promethium) crystals were synthesized using the homogeneous precipitation method; for details, see ref.^46^. Low supersaturation and increased time of the process allowed the oxalate crystals to develop to an atypical size (up to 1 mm and more, see Figure 1), while the conventional heterogeneous precipitation method gives oxalates with particle sizes only up to several microns.^42,47,48^ Harsh crystallization (hydrothermal) and drying (vacuum, elevated temperature) conditions should be avoided in order to preserve the original structure of materials, otherwise, recrystallization, polymorph formation, or even decomposition to oxides can occur.^49,50^

**Figure 2:**
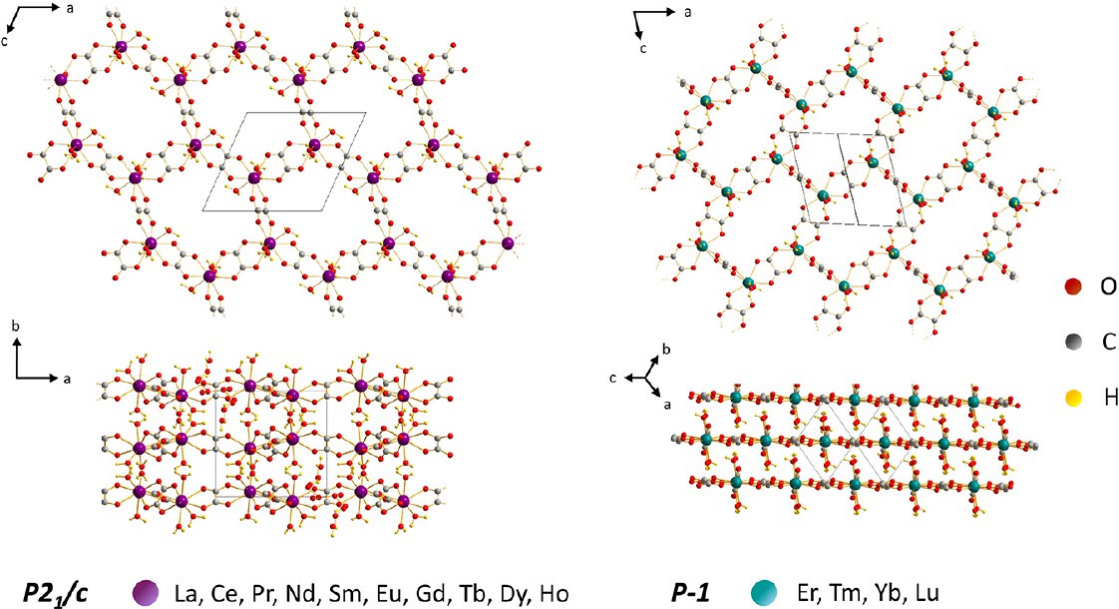
Crystal structures of trivalent lanthanide oxalates. From La to Ho (left), the compounds belong to the P21/c space group and are decahydrates, apart from Dy and Ho. The four heaviest lanthanides (Er–Lu; right) belong to the *p*-1 space group and contain six water molecules. Free water molecules in the cavities have been omitted for clarity.

The crystal structures of all the prepared lanthanide oxalates were determined. The structures of Nd, Gd, Tm, and Lu oxalates that were previously determined were confirmed by the present work. The Nd and Gd compounds were above-described decahydrate analogues in databases supplied by Ollendorff^41,51^ and Lu and Tm oxalates matched the database records of Marine Ellart^52^ and Shu-Feng Si.^44^ Works of Hansson^40^ and Wanatabe^53^ should also be mentioned, giving insight into the Er and Tm oxalate structures by processing the powder diffraction data. Up to now, the structures of Dy, Ho, Er, and Yb have not been successfully resolved or precisely described in the literature. The structures determined in this study were deposited to the Cambridge Crystallographic Data Centre under the following numbers: 2389409 to 2389414 (Lu, Tm, Dy, Yb, Er, Ho, respectively; for complete information, see Table S3). As a result of the present study, it can be stated that oxalates from La to Ho have a *P2*_*1*_*/c* structure (no. 14) of a monoclinic crystal system. From La to Tb, the oxalates are decahydrates with the unit cells comprising four Ln(III) atoms with a coordination number of 9. They can be described as catena-tris(μ_2_-oxalato-O,O’,O’’,O’’’)-hexa-aqua-di-Ln(III) tetrahydrate, simplified as [Ln_2_(C_2_O_4_)_3_(H_2_O)_6_]·4H_2_O. Each Ln(III) atom is coordinated by nine oxygens (distorted tricapped trigonal prisms), six coming from three bidentate oxalate groups and three from water molecules at almost equal distances, as seen in Figure 2 (left). Dy and Ho oxalates, both having a *P2*_*1*_*/c* structure, present a transition from decahydrates to hexahydrates. The water content was unclear from XRD analyses (significant free motion of the molecules in the cavities resulted in disordered positions in the structure). However, the thermogravimetric measurements showed that Dy oxalate is, on average, an octahydrate, and Ho oxalate is a hexahydrate (see Figure S1). The thermogravimetric curves up to 800 °C for all the compounds are available in the Supporting Information. The structure of oxalates of the last four heaviest lanthanides was analyzed in depth, and they showed a *p-1* (#2) structure. The *p-1* structure visualization is given in Figure 2 (right). Thanks to the smaller ionic radii of these heaviest lanthanides (Er to Lu), they accommodate a smaller number of water molecules in their coordination sphere. So, the systematic name to be assigned to them is catena-(bis(μ_3_-oxalato)-(μ_2_-oxalato)-tetra-aqua-di-*Ln*(III) dihydrate), simplified as [Ln_2_(C_2_O_4_)_3_(H_2_O)_4_]·2H_2_O. Each Ln atom exhibits a coordination number of 8 and is directly bound to six oxygens belonging to three coordinated oxalate anions and two more oxygens adhered to water molecules. This forms 4,4′-bicapped trigonal prism-shaped coordination polyhedra. Interestingly, for both *P2*_*1*_*/c* and *p-1* structures, two water molecules are typically trapped in each cavity, showing partial free motion. More structure visualizations are found in Figure S2.

**Figure 3:**
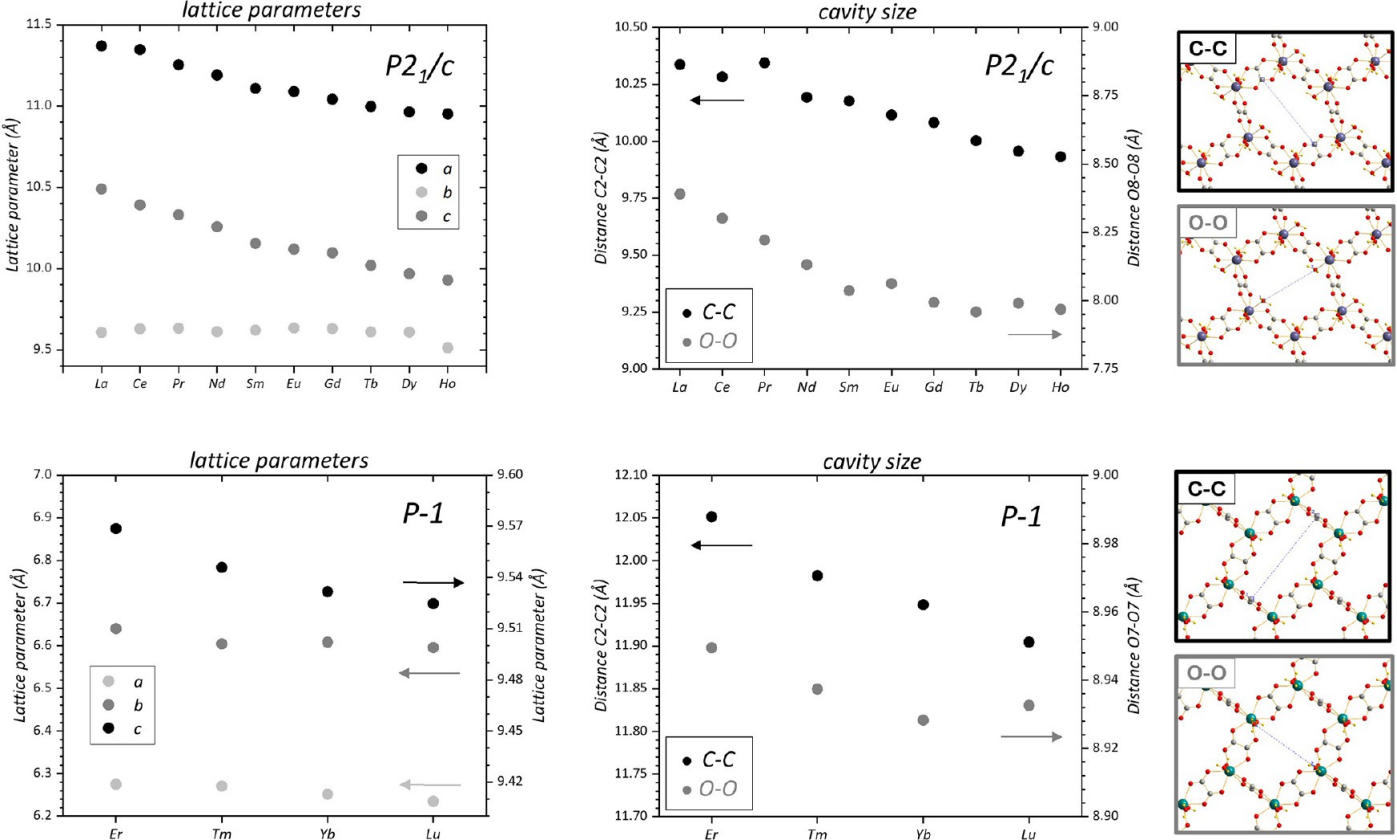
Lattice parameters and cavity sizes for the *P*2_1_/*c* (top row) and *p*-1 (bottom row) structures. The cavity size is represented by the distances of C–C and O–O atoms, as shown in the figures on the right. Results are based on single crystal XRD data.

Experimentally derived lattice parameters of all compounds are summarized in Figure 3 (left). Lanthanide contraction was evidently encoded in an almost linear decrease of lattice parameters *a* and *c*, while the *b* parameter stayed almost constant (for *P2*_*1*_*/c*). Similarly, a linear decrease was noted for parameter *c* concerning the *p-1* compounds. The lanthanide contraction was also reflected in the decreasing size of the cavity with the atomic number of the lanthanide (Figure 3 on the right). The interesting fact about the structures is that the coordination polyhedra form 2D networks that are bound together only with hydrogen bonds. For the *p-1* structure, it is more visible that the polymers cohere together, like two sides of a zip locker. Additionally, both structures contain cavities (see Figure 2), which are closed by the neighboring layers. The systematic description of the cavities concerning Ln and the type of the structure is given in Figure 3 on the right. The structure, especially for heavier lanthanides, can be activated (creation of porosities and larger surface area) by applying a vacuum and slightly elevated temperature (40 °C). Volatilization and release of water molecules led to crystal disintegration, formation of larger pores, and large surface areas of 20–40 m^2^·g^–1^, see Table S4. These values are surprisingly higher than for oxalates decomposed to nanocrystalline oxides at temperatures >500 °C, typically reaching several m^2^·g^–1^.^54,55^

### 3.2 Exfoliation of Oxalates

Liquid exfoliation of 2D materials is a simple and cost-effective method for obtaining 2D nanosheets of inorganic materials that can be later deposited as individual flakes or formed into oriented films.^56^ In recent years, exfoliation of coordination polymers was attempted by several research groups; however, a commonly wide distribution of sheet thicknesses was obtained.^57,58^ Moreover, exfoliation of many 2D materials requires toxic or high-boiling solvents (e.g., DMF, formamide), the addition of surfactants,^32,33^ and high-energy ultrasonication.^59,60^

**Figure 4:**
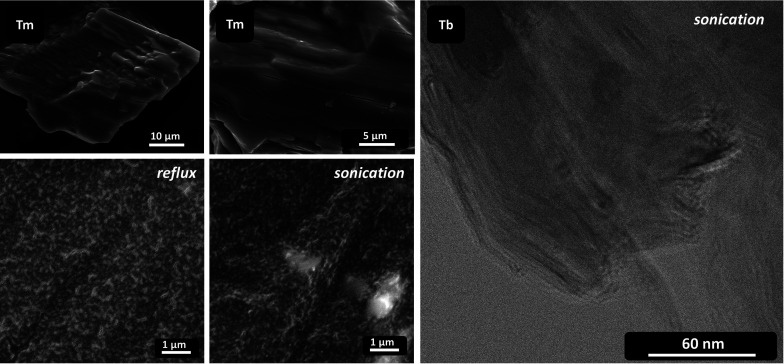
Scanning electron micrographs of Tm oxalate hexahydrate (left). The crystalline residue after partial delamination by reflux boiling in 96% EtOH shows macroscopic plane defects among the weakly bonded layers (top row). Exfoliated flakes (light gray particles < 200 nm) deposited on carbon foil after reflux and sonication in 96% ethanol (two images, bottom left). Transmission electron micrograph of Tb oxalate delaminated by sonication in 96% ethanol-agglomerated particle (right).

**Figure 5:**
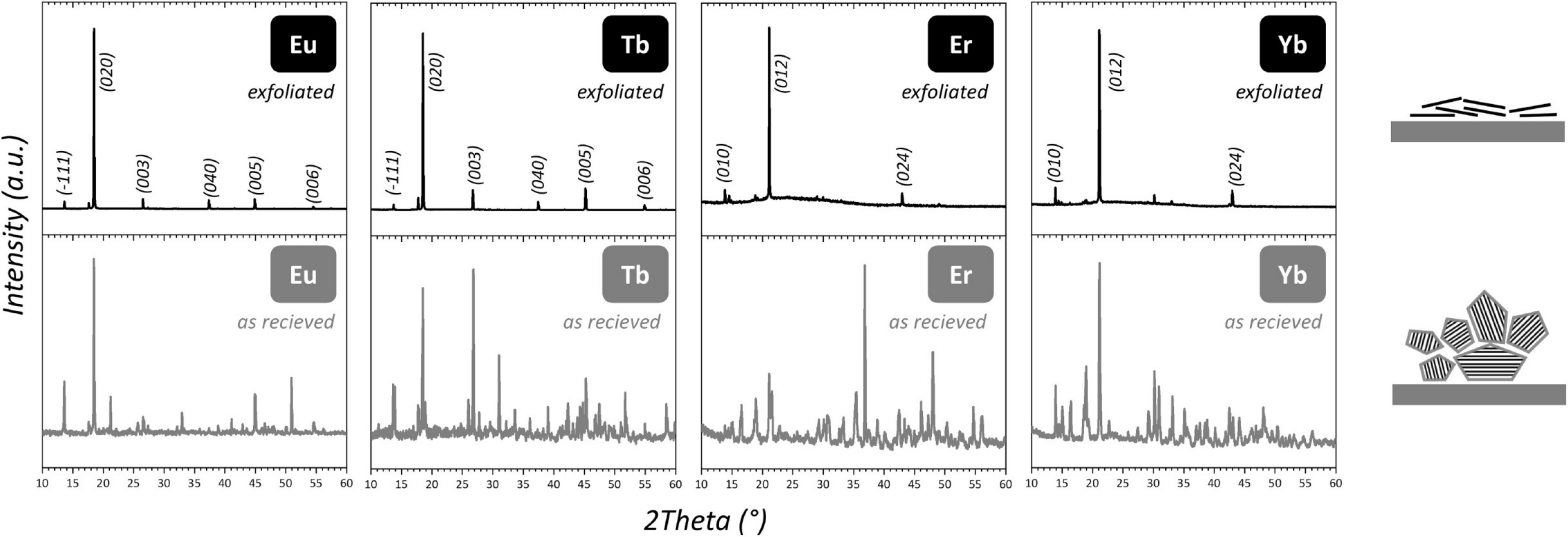
Powder X-ray diffraction of selected Ln oxalates as prepared (bottom row) and after delamination (top row) by sonication. Significant changes linked with the material’s texture in the diffraction pattern are visible. Indices of the diffraction lines are omitted for better visibility (Ln oxalates typically have >100 diffraction lines).

As all lanthanide oxalates are layered materials, they could be potentially separated into monolayers as well. No records in the open literature have indicated such behavior. We found in our large crystal samples that Ln_2_(C_2_O_4_)_3_·*n*H_2_O can be easily delaminated by sonication in a common ultrasonic bath in 96% v/v EtOH. Various solvents (MeOH, EtOH, BuOH, hexane, and formamide) have been tested, with ethanol giving the best results. Further, the ethanol purity affected the delamination results. Dry ethanol was less effective than 96% (4% water). About two h of sonication in a conventional laboratory ultrasound bath led to nanosheets of one to four layers with good reproducibility, depending on the oxalate used. Slightly worse results were obtained by refluxing in 96% EtOH; see the difference in Tm and Tb oxalate exfoliation results by scanning and transmission electron microscopy in Figure 4. The efficiency of sonication in ethanol is evident from the comparison of powder X-ray diffraction patterns of delaminated samples evaporated on a fused silica holder and parent powders (Figure 5, complete series in Figure S3). The changes in the pattern followed a strong preferential orientation of the sheets parallel with the substrate, resulting in intensifying diffractions of the planes parallel to the substrate surface and diminishing other nonbasal diffractions. This is primarily visible in the significant gain of the diffraction line intensity at 15–25°, see Figure 5. The exfoliation behavior (sonication time, dispersant, dispersion stability, etc.) should be studied more in depth. First, the exfoliation ability versus Ln ion (if any) should be assessed. Later, other possible exfoliation techniques can be explored. As for now, no systematic tendencies depending on the lanthanide in the present work were observed.

**Figure 6:**
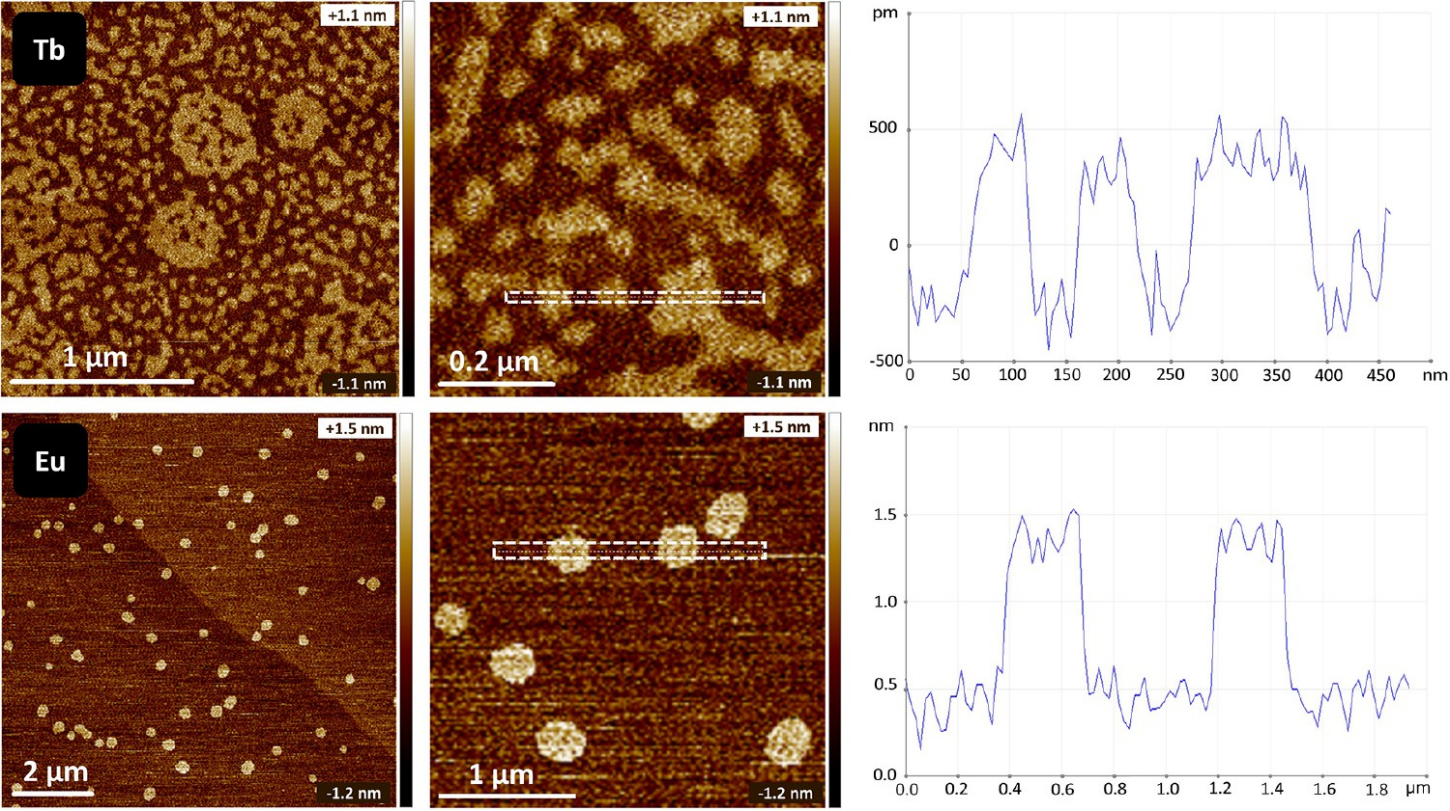
Atomic force microscopy investigations of selected samples, Tb (upper row) and Eu (bottom row) oxalates. Both were delaminated by sonication in 96% ethanol. Line measurements (right) correspond to the dashed lines in the middle figures.

The final confirmation of the exfoliation was performed by using atomic force microscopy (AFM). The AFM images for Tb and Eu oxalates exfoliated by sonication in 96% ethanol with line scans are shown in Figure 6. The AFM images of other Ln oxalates are presented in the Supporting Information. The delaminated samples consisted of nanosheets several hundred nanometers in diameter and roughly 10 Å in height. The distance between two centers of Ln atoms at the same position in the layers (derived from structural data) varies between 8.38 and 12.02 Å for Er oxalate; these values are similar for all the Ln oxalates. Therefore, the AFM images (Figure 6) indicated the presence of single-layered nanosheets. It should be noted that AFM cannot give precise measurements of layer thickness at such a low scale, partly because of the method’s limitations and partly because of the possibility of adsorbed solvents, such as discrepancies in values for graphene.^61^ As for now, the thickness of the nanosheet varied for the respective lanthanide oxalates. Thus, the Eu and Tb oxalates were exfoliated to single-layered nanosheets, whereas the Yb oxalate was exfoliated to three- to four-layered nanosheets (Figure S4). Currently, we have no explanation for why, for some oxalates, we obtained a single layer and for some not. It might be linked with the atomic number of the oxalate, drying sensitivity, or other factors. A deeper study in this direction should be performed.

### 3.3 Luminescence and Magnetic Properties

**Figure 7:**
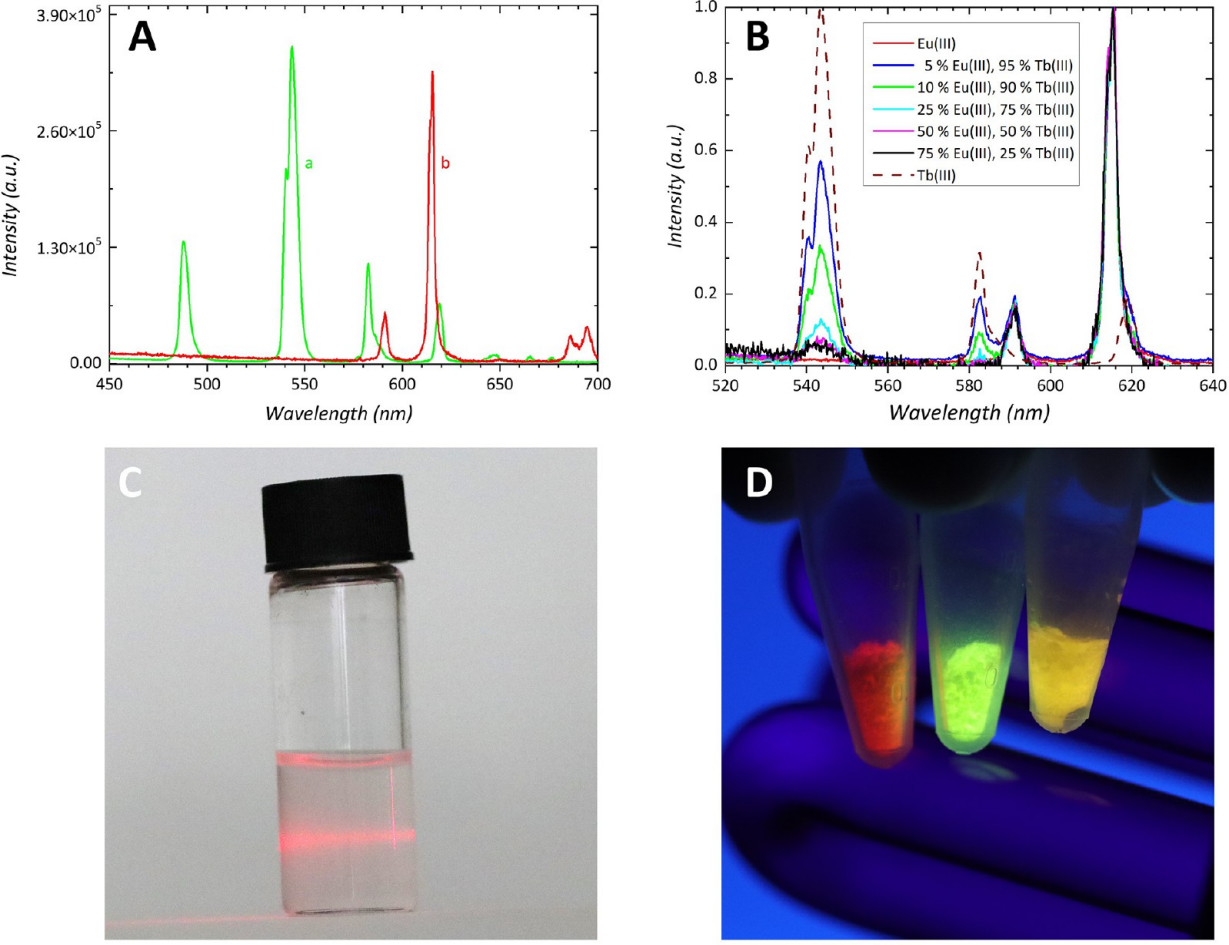
Luminescence spectra of exfoliated dry Tb oxalate (a, green) and Eu oxalate (b, red) deposited on quartz plates (A), the excitation wavelength was 350 nm. Normalized luminescence spectra of the exfoliated Eu(III) oxalate film (red) compared with those of mixed Eu(III)–Tb(III) oxalate films (B). The red emission of Eu(III) at 615 nm was used for normalization. The luminescence spectrum of the exfoliated Tb(III) oxalate film (dashed line) is added for comparison. The layers were deposited on quartz plates. Excitation wavelength was 350 nm. Exfoliation of Eu oxalate in ethanol (C)–dispersion of the nanosheets documented by the Tyndall effect , with scattering of red laser light. Luminescence of oxalate powders under UV irradiation (D), Eu oxalate (red), Tb oxalate (green), and mixed Eu–Tb (5–95) oxalate (yellow).

The basic characterizations of lanthanide-related properties were performed to outline possible applications of oxalate coordination polymers, for example, as luminescent^62−65^ or magnetic materials.^67−70^ Bulk Eu and Tb oxalates showed typical absorption spectra of Eu(III) and Tb(III) ions, corresponding to the transitions from the ^7^F_0_ and ^7^F_6_ ground states, respectively (Figure S5). The dried powders and also exfoliated Eu and Tb oxalates exhibited luminescence typical for Eu and Tb-based solid materials.^62^ For this purpose, the nanosheets were drop-cast from ethanol dispersions on quartz plates and dried. The luminescence properties of the deposited nanosheets of Tb oxalate, Eu oxalate, and mixed Tb–Eu oxalates with varying Eu content are presented in Figure 7A,B. The characteristic emission bands of Tb(III) ions in Tb oxalate, attributed to f–f transitions, were located at 488 nm (^5^D_4_–^7^F_6_ transition) in the blue region, 544 nm (^5^D_4_–^7^F_5_ transition) of the highest intensity in the green region, and at 583 and 619 nm (^5^D_4_–^7^F_4_ and ^5^D_4_–^7^F_3_ transitions, respectively). In the case of Eu oxalate, four distinct bands at 592, 615, 650 (weak), and 695 nm correspond to the transitions from ^5^D_0_ to ^7^F_*J*_ (*J* = 1–4), with the band at 615 nm having the highest intensity. The lanthanide ions can form a solid solution in the oxalate coordination polymer;^66^ therefore, mixed Eu–Tb oxalates with varying amounts of Tb (5, 10, 50, 75 mol %) were also exfoliated, and the luminescence properties of these nanosheets were also investigated. As follows from Figure 7B, the Tb(III) emissions at 544 and 583 nm decrease in intensity as the Eu(III) content increases due to the enhancement of energy transfer efficiency between Tb(III) and Eu(III) ions.

In order to compare the luminescence properties of nanosheets with those of the corresponding bulk materials and to document that exfoliation is a convenient method for the fabrication of luminescent films while keeping the original luminescence properties of bulk compounds, we recorded luminescence quantum yields (*F*_*L*_) of both oxalates. Powder and deposited exfoliated nanosheets of Eu-oxalate had a moderate *F*_*L*_ of 0.17 and 0.16, respectively. Tb-oxalate was much more emissive, with *F*_*L*_ values of 0.44 and 0.49 for powder and exfoliated nanosheets, respectively. The results indicate that exfoliated Tb and Eu oxalates keep the original luminescence intensities as bulk materials and are well-suited for the fabrication of luminescent films.

Moreover, many of the Ln complexes (including the oxalato-) are known to exhibit interesting magnetic properties, such as slow relaxation leading to single-ion magnets (SIMs)^67^ or single-molecule magnet (SMM) behavior.^68−71^ In the search for possible magnetic interactions in the coordination polymer, we performed field-dependent magnetization measurements of oxalates with Ln (Dy, Er, Tm, and Yb) having larger angular moments (Figure S6). The bulk materials exhibited paramagnetic behavior. The temperature-dependent magnetization curves (Figure S7) recorded at an applied field of 1 T (Tb) or 0.01 T (Dy–Yb) were fitted with the Curie–Weiss law, because of the present negligible interactions. The obtained Curie constants allowed for the calculation of the effective magnetic moments per atom in the measured compounds (Table S5), which have been shown to be not far off from the theoretical values. The more significant deviations were observed for Dy and Yb oxalates: Dy shows similar magnetization to the compound presented by Zhang et al.,^68^ and for the latter one, a small positive temperature-independent contribution to the magnetic susceptibility could have affected the fitting that might arise from core diamagnetism (both from the sample or the sample holder), Pauli paramagnetism, or van Vleck paramagnetism.^72^

Observation of almost no divergence between the curves performed under the zero-field-cooled and field-cooled conditions ruled out the possibility of ordered or frozen magnetic states. The susceptibility dependence of the Er and Yb samples is more complex and is influenced by thermally populated crystal field levels. However, as isothermal magnetizations measured slightly below and above the peak temperatures did not reveal any significant changes, we attribute these deviations to the resolution limits of the instrument.

## 4 Conclusions

The complete series of lanthanide oxalates, Ln_2_(C_2_O_4_)_3_·*n*H_2_O, have been synthesized, and detailed structural analysis was performed. All lanthanide oxalates showed a layered honeycomb structure; lighter lanthanides, La to Ho, crystallized in the *P2*_*1*_*/c* structure (#14), while heavier lanthanides (Er to Lu) exhibited the *P-1* (#2) structure. The total water content also followed a trend, from decahydrate for light lanthanides to hexahydrate for the four heaviest lanthanides. The sizes of the cavities in the structure were precisely determined. The size is obviously linked to the structure and lanthanide ion diameter; it ranged from 7.9–10.4 Å for *P2*_*1*_*/c* and 8.9–12.1 Å for *p-1* structures.

Importantly, we demonstrated that oxalates of heavier lanthanides, including mixed Eu–Tb oxalates, can be exfoliated to up to single-layered nanosheets, forming colloidal dispersions stable for hours without any additional stabilization. The exfoliation process can be done by sonication in a common ultrasonic bath or by reflux, all using 96% ethanol. Selected lanthanide (or mixed lanthanide) oxalates were characterized for their optical or magnetic properties.

Given the widespread use of lanthanide oxalates at the industrial level, their facile and low-cost production makes them ideal precursors for thin film fabrication. Therefore, this work can open a new R&D window in the use of lanthanide oxalates, but not solely, in 2D materials. They can be used on their own but also in combination with other 2D nanosheets.

## Supporting Information Available

Summary of crystallographic characteristics, thermogravimetric measurements for Er, Tm, Yb, Lu oxalates; specific surface measurements (all samples), powder -Xray diffractograms (all samples), Atomic force microscopy of delaminated samples, spectral (Tb, Eu) and magnetic properties (Dy, Er, Tm, Yb) (PDF)
