# Supplementary material for: Lanthanide Oxalates: From Single Crystals to 2D Functional Honeycomb Nanosheets
Source: Inorg Chem. 2025 Feb 18;64(8):3686–95. doi: 10.1021/acs.inorgchem.4c04293 (PMC11881034; doi:10.1021/acs.inorgchem.4c04293)
Supplement: Supplementary file 1 — ic4c04293_si_001.pdf [file ic4c04293_si_001.pdf]

## Supplementary information

# Lanthanide oxalates: from single crystals to 2D functional honeycomb nanosheets

**Daniela Vreonika Uríková[1], Giannis Kampitakis[2], Ivana Císařová[1], Adam Alemayehu[2], Matouš Kloda[2], Dominika Zákutná[1], Kamil Lang[2], Jan Demel[2], Václav Tyrpekl[1,*]**

*1. Department of Inorganic Chemistry, Faculty of Science, Charles University, Hlavova, 2030 Prague, Czech Republic*

*2. Institute of Inorganic Chemistry of the Czech Academy of Sciences, 250 68, Husinec-Řež 1001, Czech Republic*

**Corresponding author: vaclav.tyrpekl@natur.cuni.cz*

**Information about the used software**

The graphs were plotted using the OriginPro software 2021 (Copyright © 1991-2020 OriginLab Corporation).

The structures were visualised using the Diamond (Version 4.6.8, Copyright © 1997-2022 Crystal Impact GbR, Bonn, Germany) or the Vesta software (https://jp-minerals.org/vesta/en/).

The powder diffraction data was treated using Jana 2006 (Petříček V, Dušek M, Palatinus L. Crystallographic computing system JANA2006: General features. Zeit Krist Cryst Mater. 2014; 229(5): 345–52. https://doi.org/10.1515/zkri-2014-1737) or the FullProf package (Rodriguez-Carvajal J. Fullprof Program. Phys B., 1993; 192: 55.).

The single crystal X-ray diffraction data were solved using the SHELXT 201464 program (G. M. Sheldrick, Acta Crystallogr. Sect. A: Found. Crystallogr., 2015, 71, 3–8.) and refined using the SHELXL 2017 (M. Sheldrick, Acta Crystallogr. Sect. C: Struct. Chem., 2015, 71, 3–8.).

**Table S1 Lattice parameters of Ln oxalates according to Hansson[1].**

| Ln | *a* [Å] | *b* [Å] | *c* [Å] | α [°] | β [°] | γ [°] |
|---|---|---|---|---|---|---|

| | | | | | | |
|---|---|---|---|---|---|---|
| Ce | 11,780(3) | 9.625(3) | 10.401(3) | 90.00 | 119.07(2) | 90.00 |
| Nd[2] | 11.900 | 9.880 | 10.400 | 90.00 | 119.00 | 90.00 |
| Nd[3] | 11.678(2) | 9.652(2) | 10.277(2) | 90.00 | 118.92(2) | 90.00 |
| Sm | 11.577(2) | 9.643(2) | 10.169(2) | 90.00 | 118.87(2) | 90.00 |
| Gd | 11.516(2) | 9.631(3) | 10.081(3) | 90.00 | 118.82(2) | 90.00 |
| Dy | 11.433(2) | 9.615(3) | 9.988(3) | 90.00 | 118.76(2) | 90.00 |
| Ho | 11.393(2) | 9.607(3) | 9.955(3) | 90.00 | 118.75(2) | 90.00 |
| Er (10) | 11.359(2) | 9.616(2) | 9.940(2) | 90.00 | 118.72(1) | 90.00 |
| Er (6) | 9.644(2) | 8.457(3) | 9.836(3) | 93.54(1) | 105.99(1) | 85.05(3) |
| Tm | 9.620(3) | 8.458(3) | 9.808(3) | 93.44(1) | 106.12(1) | 85.13(3) |
| Yb | 9.611(2) | 8.457(2) | 9.778(2) | 93.39(1) | 106.24(1) | 85.29(2) |
| Lu | 9.597(3) | 8.455(2) | 9.758(3) | 93.42(1) | 106.27(1) | 85.41(2) |

**Table S2 Lattice parameters of Ln oxalates according to Ollendorff[4].**

| Ln | *a* [Å] | *b* [Å] | *c* [Å] | α [°] | β [°] | γ [°] |
|---|---|---|---|---|---|---|
| La | 11.370 | 9.608 | 10.490 | 90.00 | 114.57 | 90.00 |
| Ce | 11.347 | 9.630 | 10.392 | 90.00 | 114.52 | 90.00 |
| Pr | 11.254 | 9.633 | 10.331 | 90.00 | 114.52 | 90.00 |
| Nd | 11.191 | 9.612 | 10.257 | 90.00 | 114.42 | 90.00 |
| Sm | 11.108 | 9.621 | 10.155 | 90.00 | 114.32 | 90.00 |
| Eu | 11.089 | 9.635 | 10.120 | 90.00 | 114.25 | 90.00 |
| Gd | 11.042 | 9.631 | 10.097 | 90.00 | 114.14 | 90.00 |
| Tb | 10.997 | 9.611 | 10.020 | 90.00 | 114.11 | 90.00 |
| Dy | 10.964 | 9.610 | 9.968 | 90.00 | 114.09 | 90.00 |

**Discussion on the water content in Ln oxalates**

Even though Ln oxalates have been quite well known for a long time, the information concerning their water content and their thermal decomposition seem to come about as rather uncertain and contradictory. From a purely logical point of view, it can be presumed that the larger the metal cation from this isomorphous series the easier it accommodates more water molecules. Although, they would be less strongly bonded, particularly those coordinated directly to the metal. The other side of the coin is that a small lattice of heaviest lanthanides could sterically exclude the lodging of ten water molecules.

For the lighter part of the series, the majority of the authors agree on a hydration number of around 10 with only small deviations like, e.g., Michaelides[5] stating 9.5 for La oxalate or Hansson's 10.5 for Nd oxalate[5]. Older literature sources[6] claim that the variation between 9.6 to 10.9 mols of water per mol of oxalate is found for hydrates produced and isolated by usual methods. Alternatively, the work of Lowenstein[7] indicates that compounds previously classed at 9-, 10-, and 11-hydrates are to be regarded as interstitial hydrates, to which no definite composition can be attributed. But it is unclear whether several other substances containing considerably more water, are to be included in this category or rather be regarded as separate entities[7]. It is for example the case of 14-hydrate of Wirth (Er), 12-hydrate of Ping Wang (Pr) or 18-hydrates of Watanabe (Ho, Er, Tm, Yb). To the matter of the latter one, the publication of Camara[8] might shed some light. It was stated that the 18-hydrate of Er was prepared within the work and its structure was resolved as well – it showed to be a trigonal crystal class compound. Watanabe possibly encountered a similar substance.

Besides these, the heaviest lanthanides show a stable preference for the hexahydrate form. In the literature Tm to Lu oxalates are (with sparse exceptions) reported as 6-hydrates, even when they settle in different structures[9,10,11]. Exceptions are stated for Yb, coming as tetragonal 3-hydrate in the work of Prozorovskii[12] or 8-

hydrate product in the study of Ping Wang. 5-hydrates of Tm and Yb observed by Wendlandt[13] are by later literature considered to correspond to 6-hydrates.

Three of the Ln, Dy, Ho and Er are especially diverse concerning hydration numbers, they form an intermediary between the 10-hydrate and the 6-hydrate series. Ollendorff, Hansson and Watanabe claim only the existence of 10-hydrate for Dy compound, whereas Dy 8- and 8.5-hydrates were later synthesised by S. Zhang[14] and Y. Zhang[15]. In the latter case, the structure measured by means of the X-ray synchrotron beamline and refined to detail agreed well with the 10-hydrate of Ollendorff.

Hansson and Watanabe also agree on the formation of both 6- and 10-hydrates for Er and Ho, with Hansson stating, that Ho oxalate 6-hydrate comes never as a pure monophasic compound. Formation of Er oxalate 10-hydrate was confirmed by Camara[9] and observed too in the work of Kalinina[16] – this structure agreed more with Watanabe's record. Moreover, Er 3-hydrates were prepared by Ellart[11] and Steinfink[17], the former belonging to the monoclinic crystal system, the other to the tetragonal.

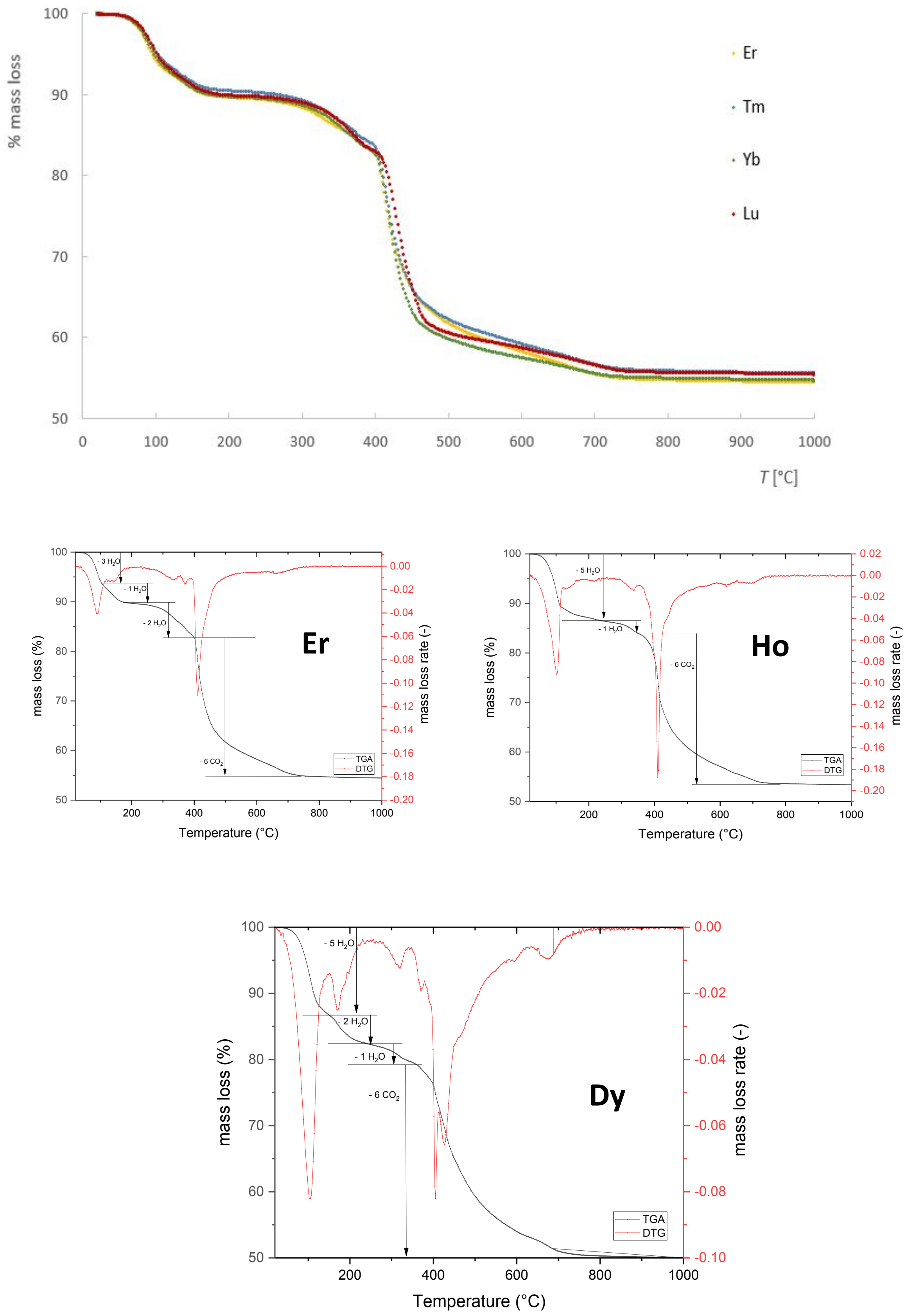


**Figure S1 TG curves of selected Ln oxalate hydrates.**

**TGA curves for all the La oxalate samples prepared in the study:**

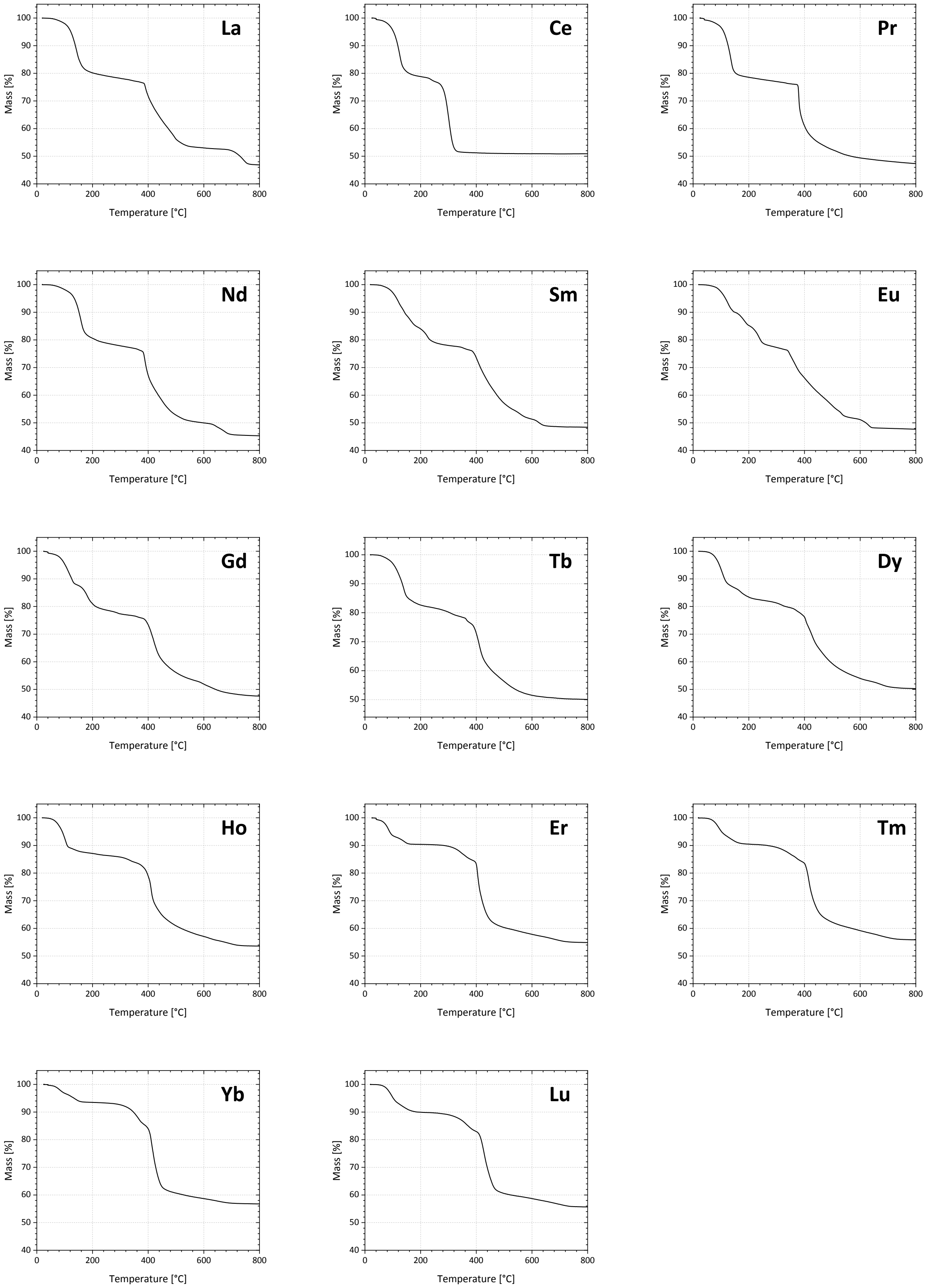

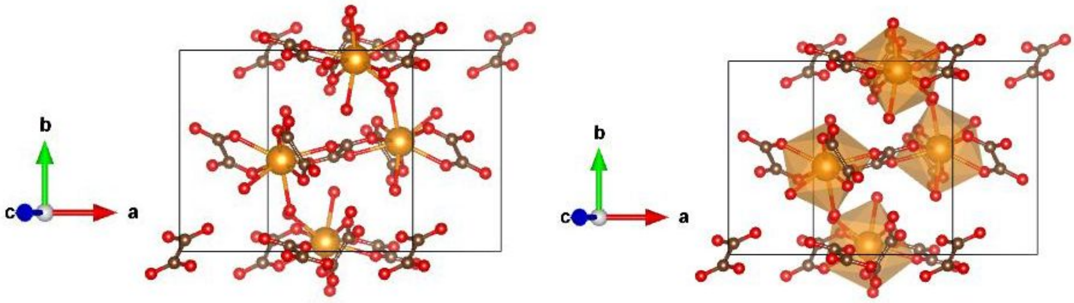


Unit cell of Nd oxalate 10-hydrate. Ball and stick model (right), coordination polyhedra (left).

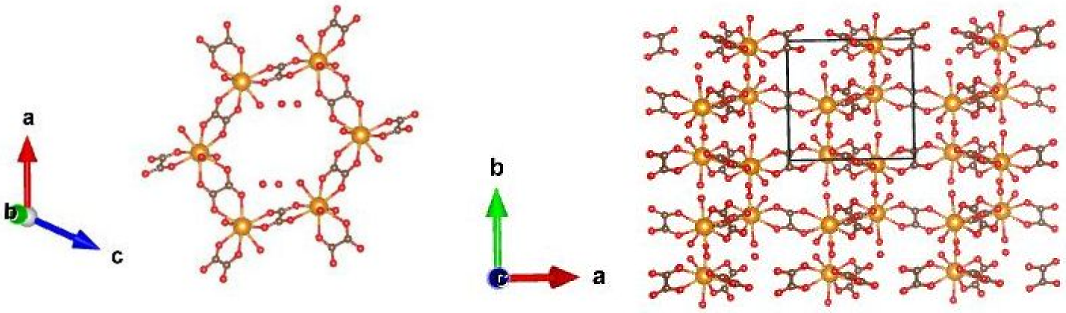


Nd oxalate hexagonal ring sub-structure. Viewed from the direction of crystallographic vector b (right). Nd oxalate layered structure viewed from the direction of crystallographic vector c (right).

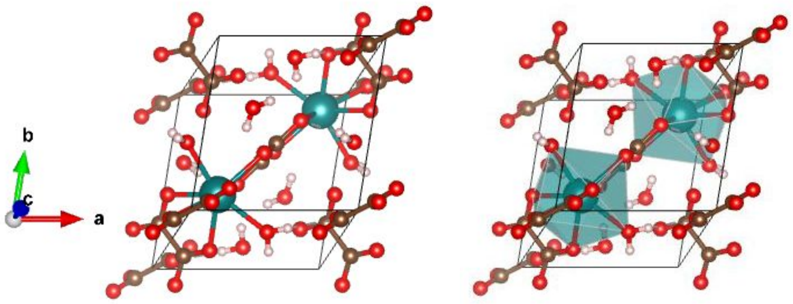


Unit cell of Er oxalate 6-hydrate. Ball and stick model (right), coordination polyhedra (left).

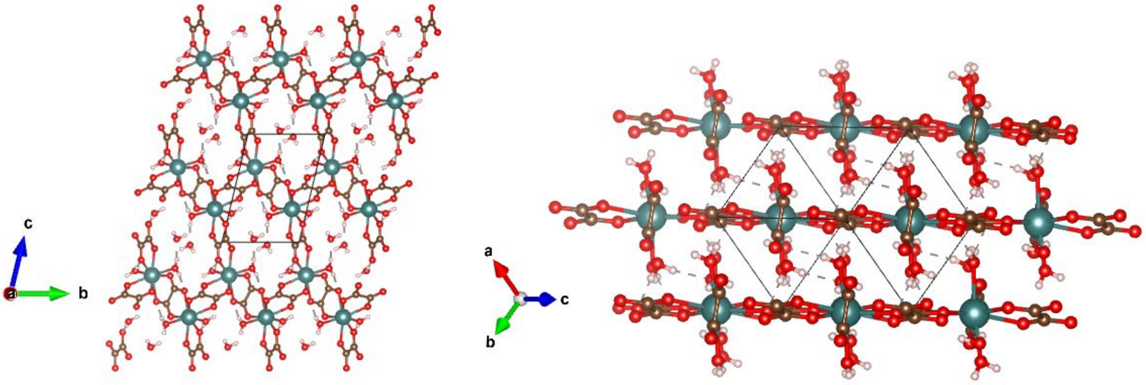


Er oxalate structure, viewed from the direction of crystallographic vector a (right). Er oxalate layered 3D structure, viewed from the direction of (111) plane (left).

**Figure S2 Visualizations of both Ln oxalates structures using Vesta software.**

**Table S3. Crystal data, data collection, and refinement parameters.**

| **Compound** | **dyox** | **(hoox)** | **(erox)** |
|---|---|---|---|
| CCDC | 2389411 | 2389414 | 2389413 |
| Formula[d] | $C_3H_6DyO_9{\cdot}(O_{1.06}){\cdot}H_2O$ | $C_3H_6HoO_9{\cdot}2H.$ 2.002(O) | $C_3H_4ErO_8{\cdot}H_2O$ |
| M.w.[d] | 383.56 | 385.02 | 353.34 |
| Crystal system<br>Space group | Monoclinic<br>$P2_1/c$ (No. 14) | Monoclinic<br>$P\ 2_1/c$ (No.14) | Triclinic<br>$P$-1 (No. 2) |
| $a$ [Å] | 10.9884 (2) | 10.9517 (4) | 6.2749 (2) |
| $b$ [Å] | 9.5290 (3) | 9.5129 (3) | 6.6401 (3) |
| $c$ [Å] | 9.9460 (3) | 9.9292 (4) | 9.5687 (4) |
| $\alpha$ [°] | | | 75.379 (1) |
| $\beta$ [°] | 114.451 (1) | 114.462 (1) | 81.135 (1) |
| $\gamma$ [°] | | | 80.854 (1) |
| $Z$ | 4 | 4 | 2 |
| $V$[Å$^3$] | 948.03 (5) | 941.59 (6) | 378.15 (3) |
| Temperature | 120 | 120 | 120 |
| $D_x$ [g cm$^{-3}$] | 2.687 | 2.716 | 3.103 |
| Crystal size [mm] | 0.06 × 0.06 × 0.03 | 0.11 × 0.06 × 0.05 | 0.10 × 0.07 × 0.05 |
| Crystal color, shape | prism, colourless | prism, pink | prism, pink |
| μ [mm$^{-1}$] | 7.93 | 8.45 | 11.12 |
| $T_{min}$,$T_{max}$ | 0.66, 0.78 | 0.66, 0.69 | 0.49, 0.62 |
| Measured reflections | 22789 | 74720 | 8766 |
| Independent diffractions ($R_{int}$[a]) | 2736, (0.033) | 2756, (0.040) | 1729, (0.026) |
| Observed diffract. [I>2σ(I)] | 2607 | 2587 | 1696 |
| No. of parameters | 155 | 155 | 118 |
| $R$[b] | 0.016 | 0.014 | 0.012 |
| $wR(F^2)$ for all data | 0.033 | 0.032 | 0.029 |
| GOF[c] | 1.18 | 1.22 | 1.08 |
| Residual electron density [e/Å$^3$] | 0.62, −0.56 | 0.70, −0.60 | 0.87, −0.75 |

[a]$R_{int} = \Sigma|F_o^2 - F_{o,mean}^2|/\Sigma F_o^2$; [b]$R(F) = \Sigma||F_o| - |F_c||/\Sigma|F_o|$; $wR(F^2) = [\Sigma(w(F_o^2-F_c^2)^2)/(\Sigma w(F_o^2)^2)]^{½}$;

[c]$GOF = [\Sigma(w(F_o^2-F_c^2)^2)/(N_{diffrs}-N_{params})]^{½}$; [d]Formula for **dyox** and **hoox** is the result of the inclusion of disordered, partially presented water molecules.

**Table S3 (continuation) Crystal data, data collection, and refinement parameters.**

| **Compound** | **tmox** | **ybox** | **(luox)** |
|---|---|---|---|
| CCDC | 2389410 | 2389412 | 2389409 |
| Formula | $C_3H_4O_8Tm{\cdot}H_2O$ | $C_3H_4O_8Yb{\cdot}H_2O$ | $C_3H_4LuO_8{\cdot}H_2O$ |
| M.w. | 355.01 | 359.12 | 361.05 |
| Crystal system<br>Space group | Triclinic<br>*P*-1(No. 2) | Triclinic<br>*P* -1 (No. 2) | Triclinic<br>*P*-1 (No. 2) |
| *a* [Å] | 6.2707 (4) | 6.2517 (4) | 6.2348 (2) |
| *b* [Å] | 6.6043 (4) | 6.6083 (4) | 6.5955 (3) |
| *c* [Å] | 9.5459 (6) | 9.5317 (6) | 9.5247 (4) |
| *α* [°] | 75.177 (2) | 75.296 (2) | 75.284 (2) |
| *β* [°] | 81.088 (2) | 80.808 (2) | 80.636 (2) |
| *γ* [°] | 81.122 (2) | 81.220 (2) | 81.288 (2) |
| *Z* | 2 | 2 | 2 |
| $V [Å^3]$ | 374.83 (4) | 373.40 (4) | 371.28 (3) |
| Temperature | 120 | 120 | 120 |
| $D_x$ [g $cm^{-3}$] | 3.145 | 3.194 | 3.230 |
| Crystal size [mm] | 0.08 × 0.07 × 0.04 | 0.07 × 0.04 × 0.03 | 0.03 × 0.03 × 0.03 |
| Crystal color, shape | prism, colourless | prism, colourless | prism, colourless |
| μ [$mm^{-1}$] | 11.86 | 12.55 | 13.32 |
| $T_{min}$,$T_{max}$ | 0.52,0.64 | 0.54,0.69 | 0.57, 0.68 |
| Measured reflections | 53076 | 31039 | 12503 |
| Independent diffractions ($R_{int}{}^a$) | 1718, (0.034) | 2179, (0.037) | 1698, (0.040) |
| Observed diffract. [I>2σ(I)] | 1690 | 2150 | 1608 |
| No. of parameters | 118 | 119 | 118 |
| $R^b$ | 0.009 | 0.010 | 0.015 |
| $wR(F^2)$ for all data | 0.021 | 0.023 | 0.030 |
| GOF$^c$ | 1.09 | 1.09 | 1.03 |
| Residual electron density [e/$Å^3$] | 0.80, −0.62 | 1.00, −0.89 | 0.77, −0.65 |

$^aR_{int} = \Sigma|F_o^2 - F_{o,mean}^2|/\Sigma F_o^2$; $^bR(F) = \Sigma||F_o| - |F_c||/\Sigma|F_o|$; $wR(F^2) = [\Sigma(w(F_o^2 - F_c^2)^2)/(\Sigma w(F_o^2)^2)]^{½}$;

$^c\mathrm{GOF} = [\Sigma(w(F_o^2 - F_c^2)^2)/(N_{diffrs} - N_{params})]^{½}$;

**Table S4 Specific surface measurements ($N_2$ sorption) of oxalates of heavier lanthanides after activation at 40 °C for 24 hours in vacuum.**

| Ln(III) oxalate | S (BET) [$m^2/g$] | S(BJH) [$m^2/g$] | Average pore size [nm] |
|---|---|---|---|
| Eu | 27 | 30 | 7.6 |
| Gd | 43 | 54 | 5.0 |
| Tb | 20 | 27 | 8.5 |
| Dy | 39 | 44 | 8.5 |
| Ho | 32 | 40 | 10.6 |
| Er | 26 | 32 | 11.8 |
| Tm | 22 | 27 | 7.8 |
| Yb | 32 | 39 | 5.6 |
| Lu | 31 | 40 | 5.5 |

**Figure S3 X-ray powder diffractiograms of various oxalate samples (below).**

La

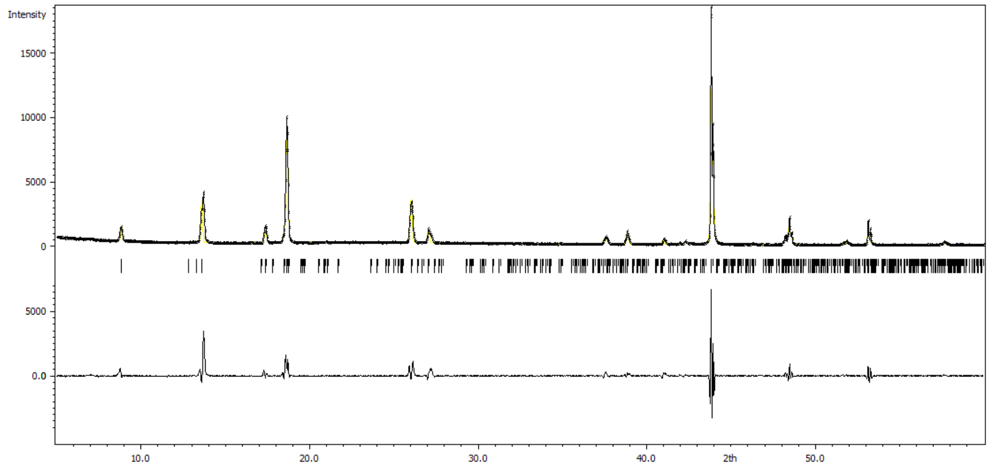


Ce

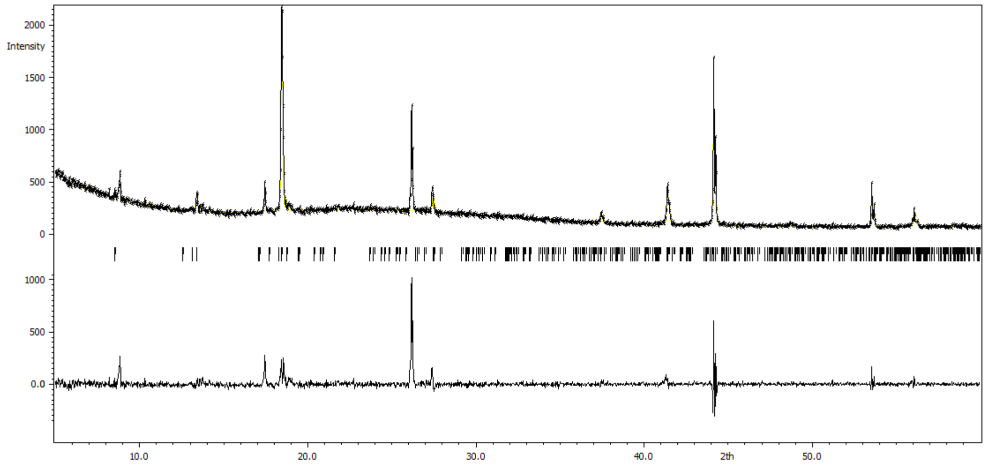


Diffractograms of Ln oxalates: La and Ce, followed by the rest of the series, marked respectively.

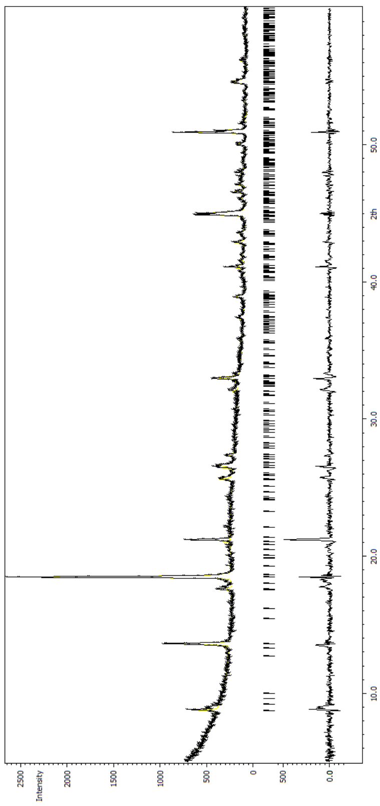

Eu

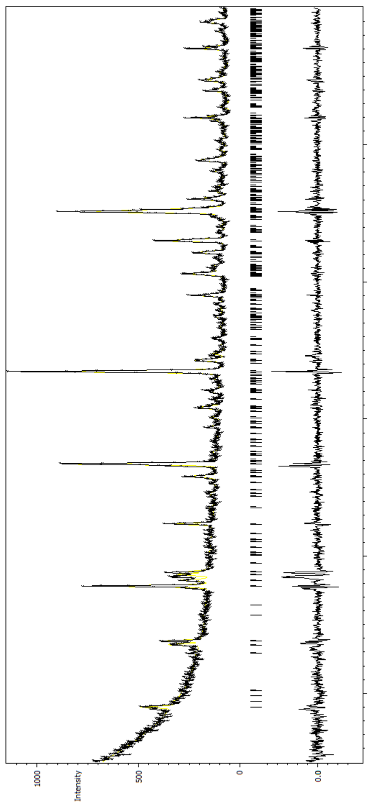

Gd

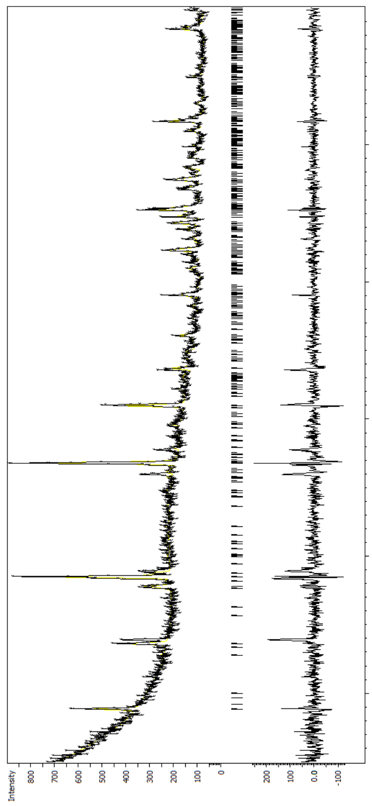

Tb

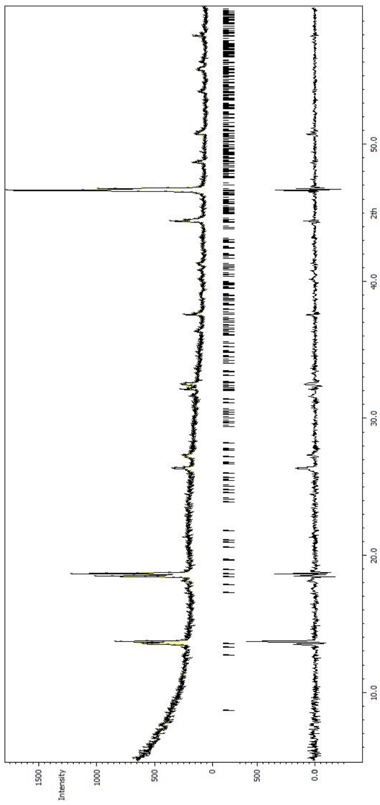

Pr

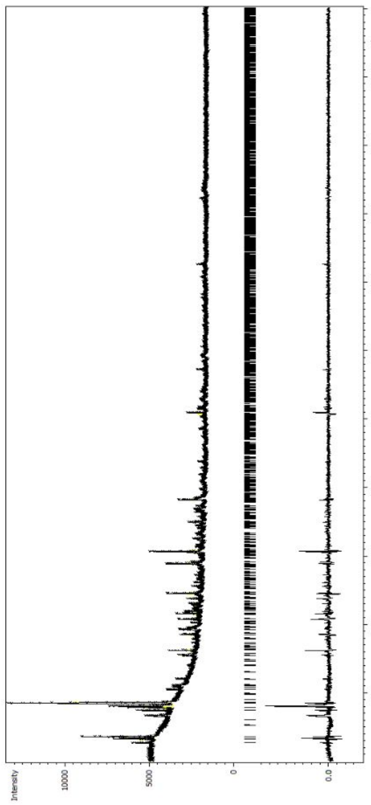

Nd

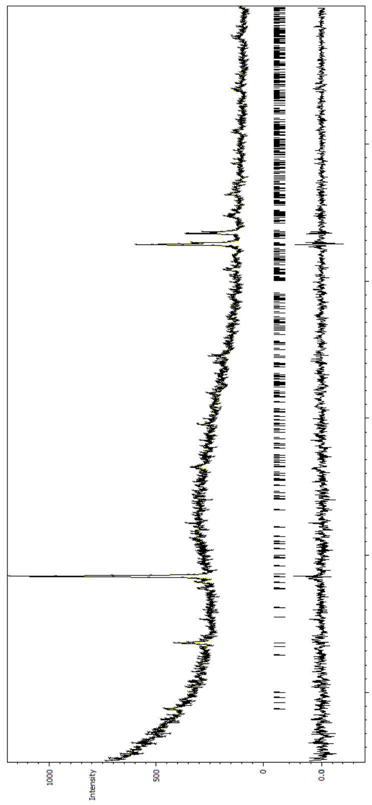

Sm

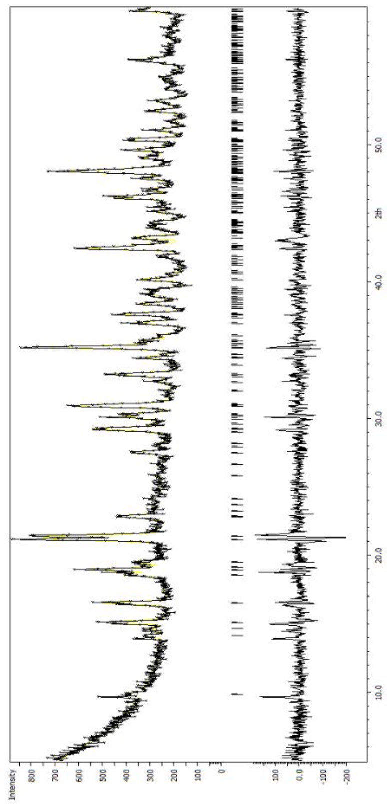

Tm

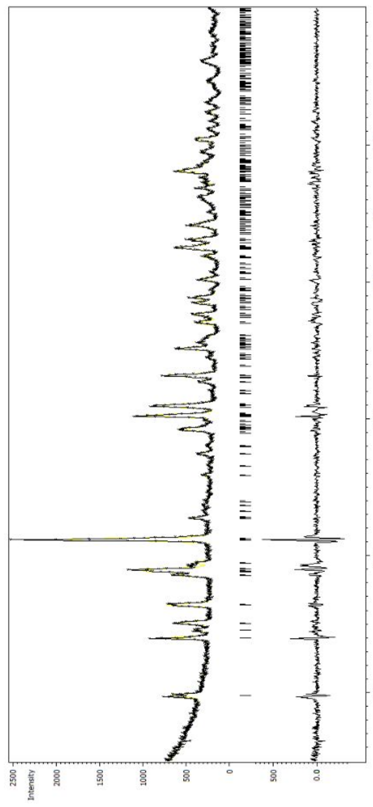

Yb

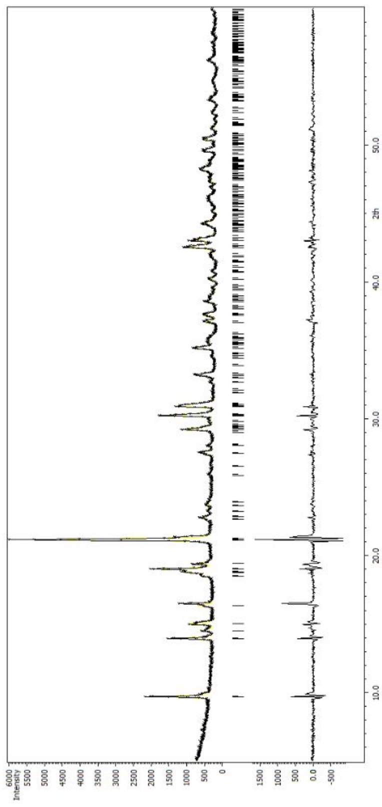

Lu

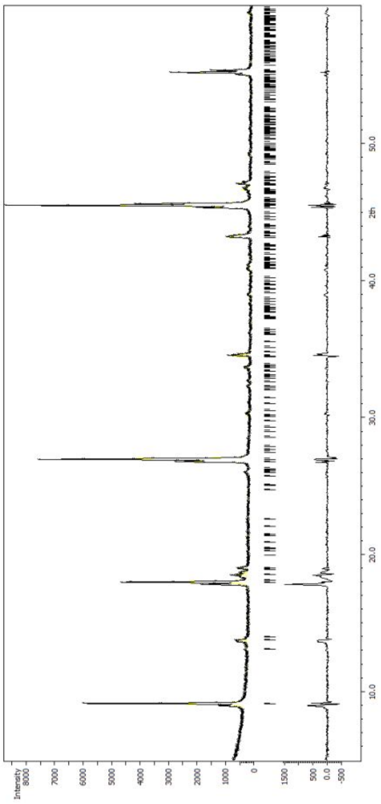

Dy

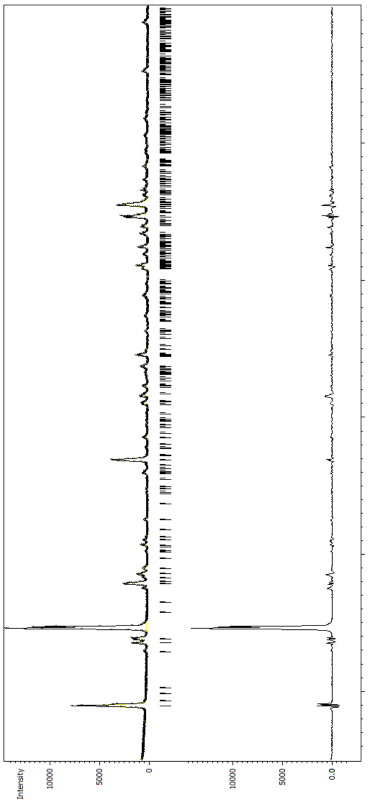

Ho

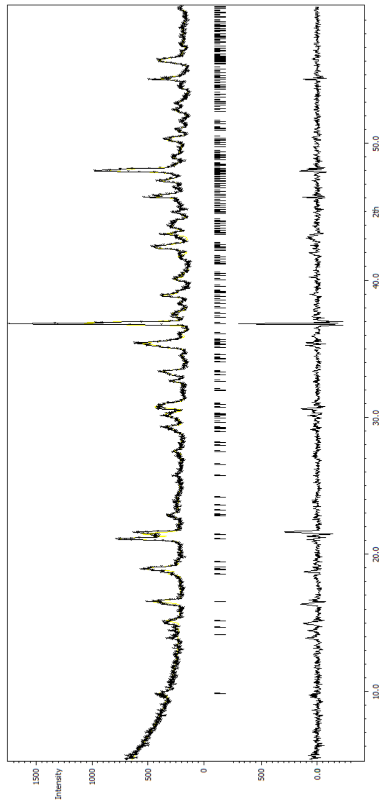

Er

**Powder X-ray diffraction of delaminated samples (below)**

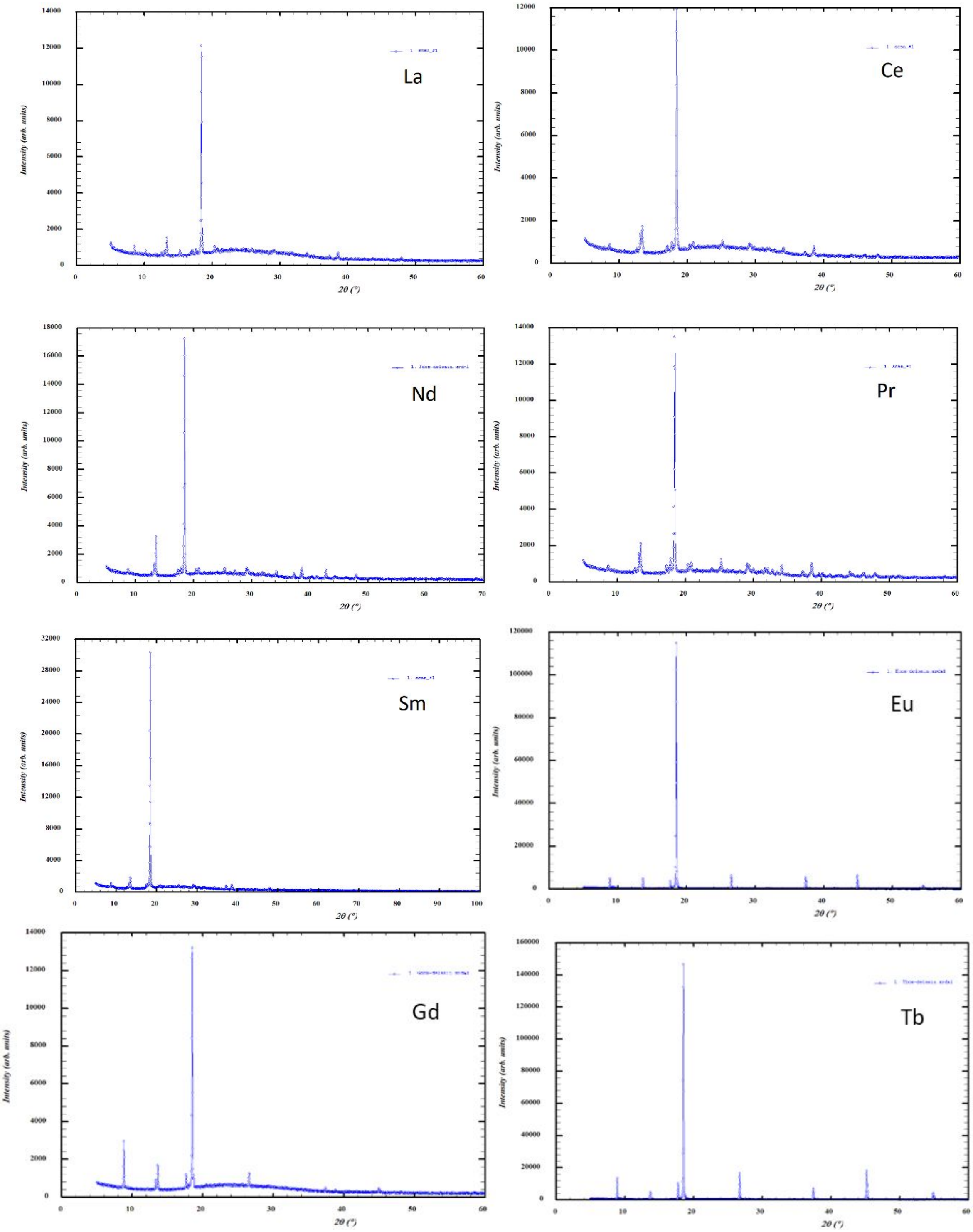

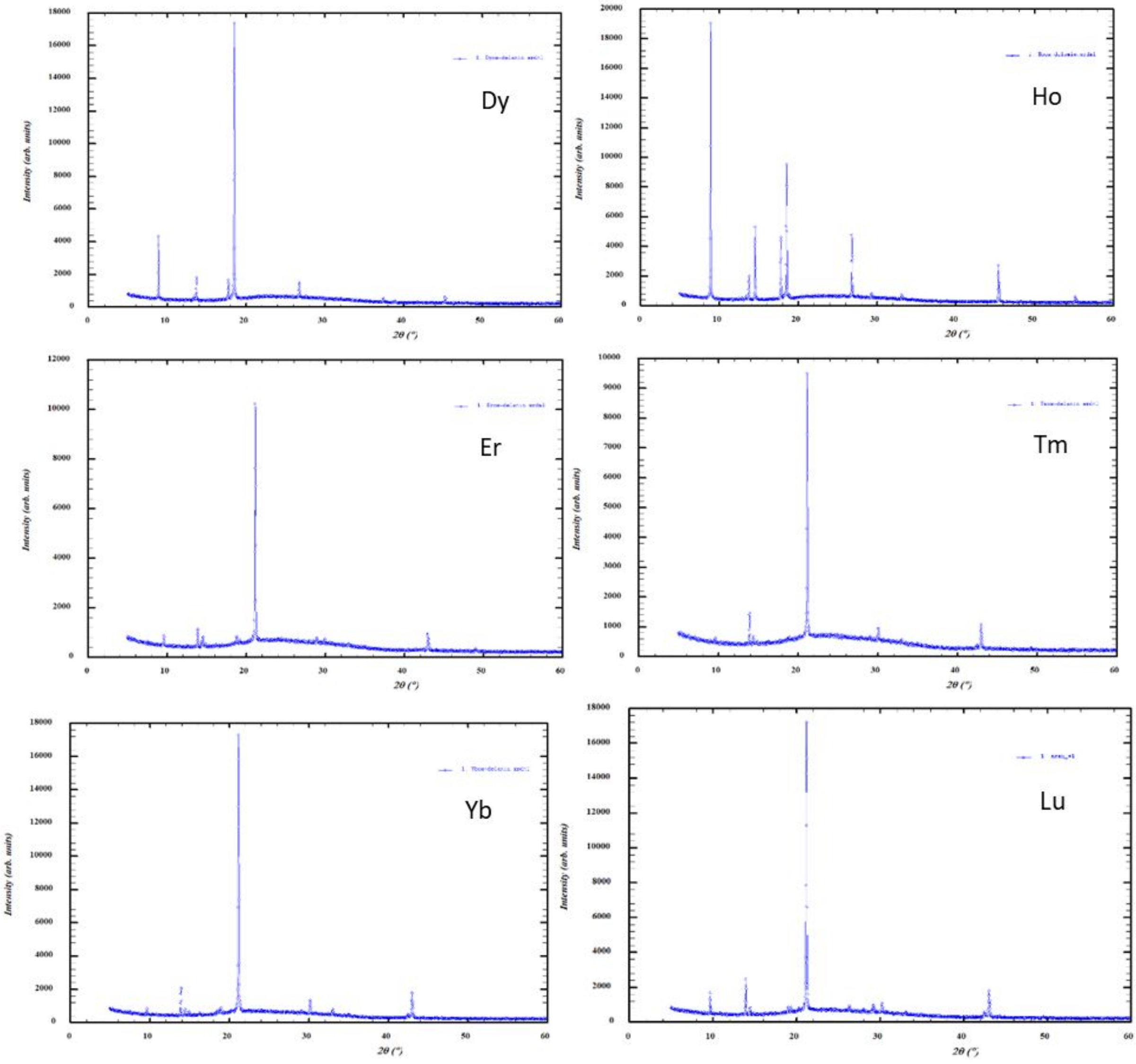


Diffractograms of delaminated Ln oxalates: Eu, Gd, Tb, Dy, Ho, Er, Tm, Yb and (down) Lu, marked respectively.

**Figure S4 Additional delamination results by atomic force microscopy (below).**

Delamination for the purposes of AFM measurement was done as a standardised procedure: synthesised Ln oxalate was poured over by ethanol 96 % - preserving the ratio of 1 mg per 1 l of ethanol and sonicated for 2 hours. The dispersion was let to rest for about 10 minutes.

For AFM measurement 80μL of the delaminated oxalate dispersion was placed on the freshly cleaved mica surface using spin coating method. Measurements were done for the oxalates of Eu, Tb, Er, tm, Yb and Lu. Terbium Oxalate + 96% EtOH 26ml (1mg/ml) - *2hours sonication*

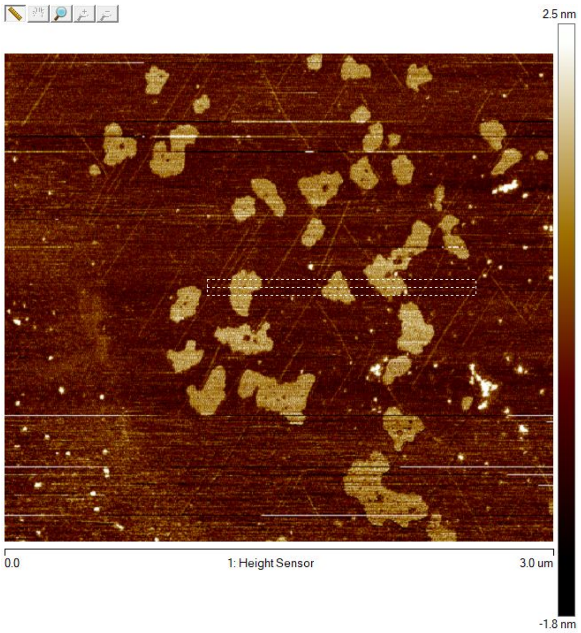


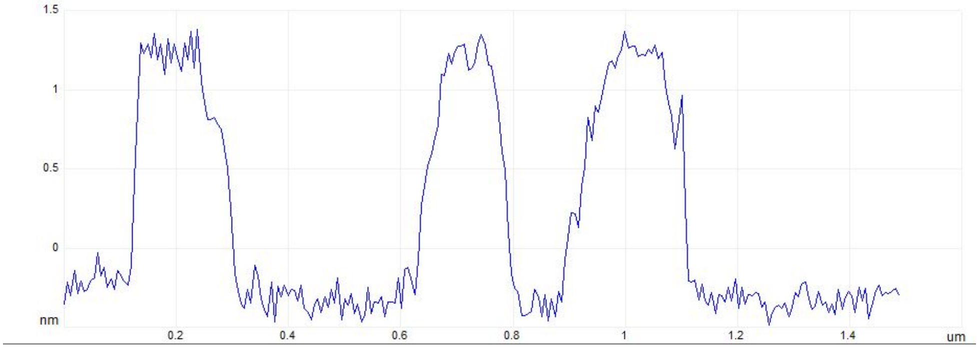


Height: around 1.5nm; Horizontal distance: 50-400nm

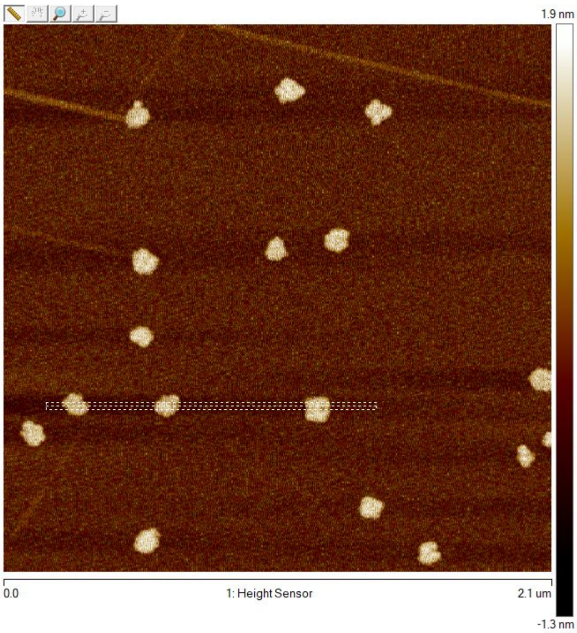


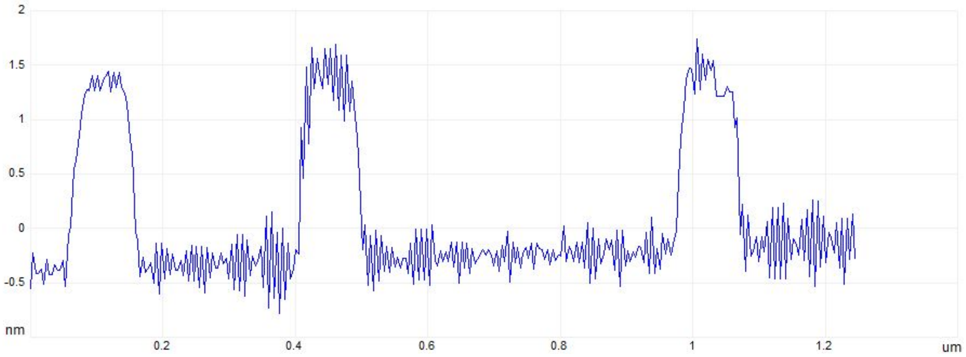


Height: around 1.5-2nm; Horizontal distance: around 100nm

Terbium Oxalate + 96% EtOH 12ml (1mg/ml) - *2hours sonication*

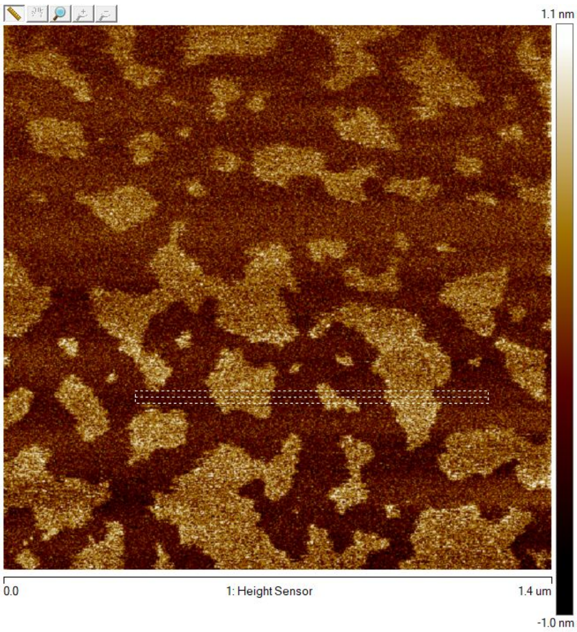

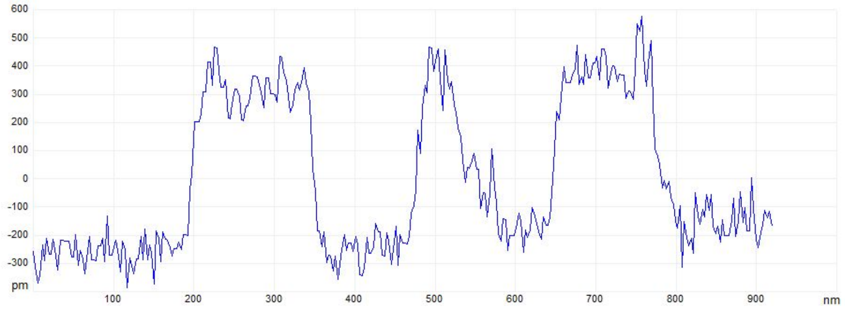


Height: around 0.7nm; Distance: 30-400nm

Terbium-Europium (95%-5%) Oxalate + 96% EtOH 11ml (1mg/ml) - *2hours sonication*

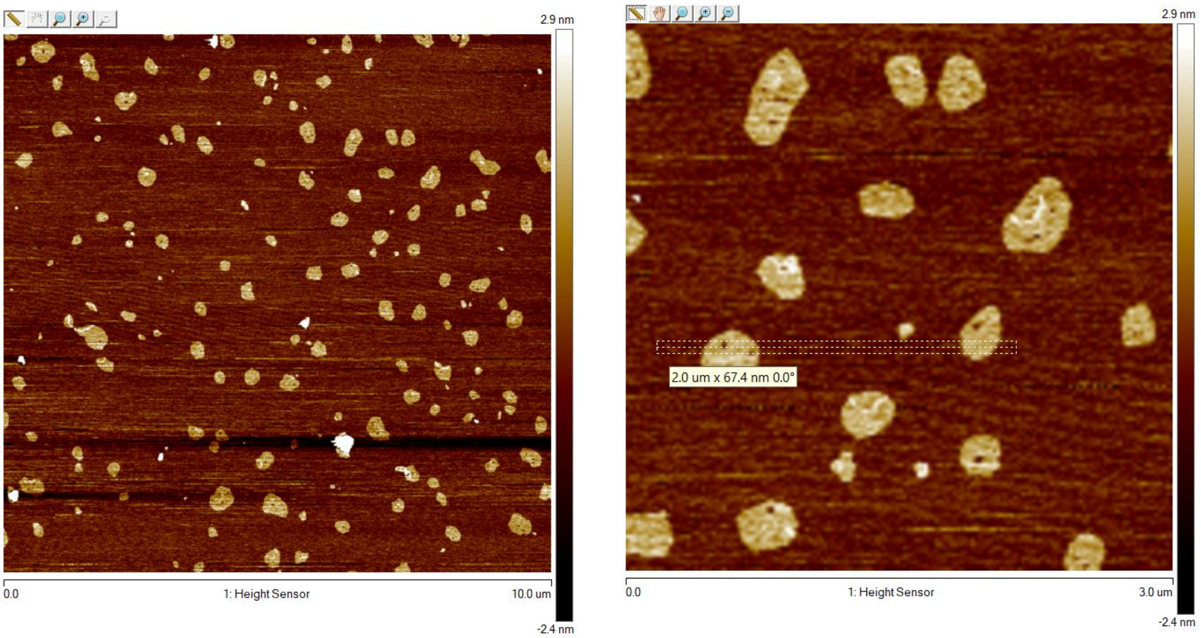


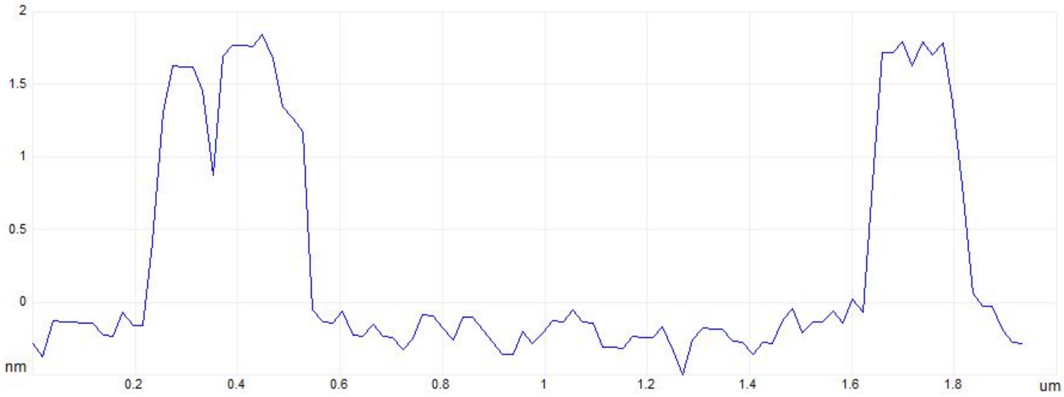


Height: around 2 nm; Horizontal distance: 100-400nm

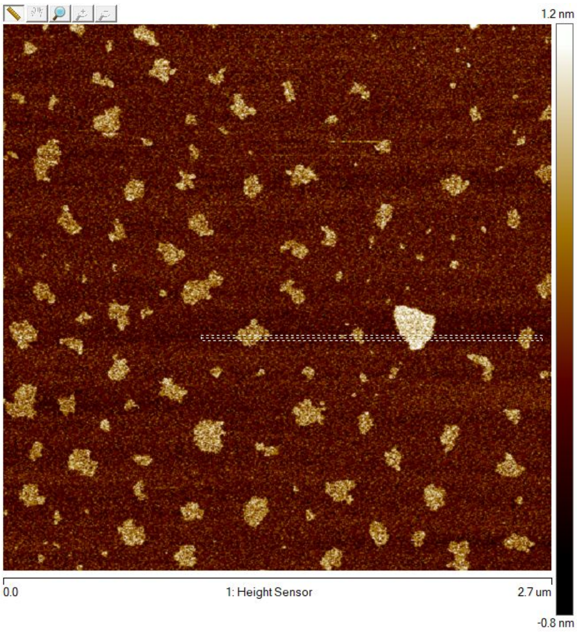


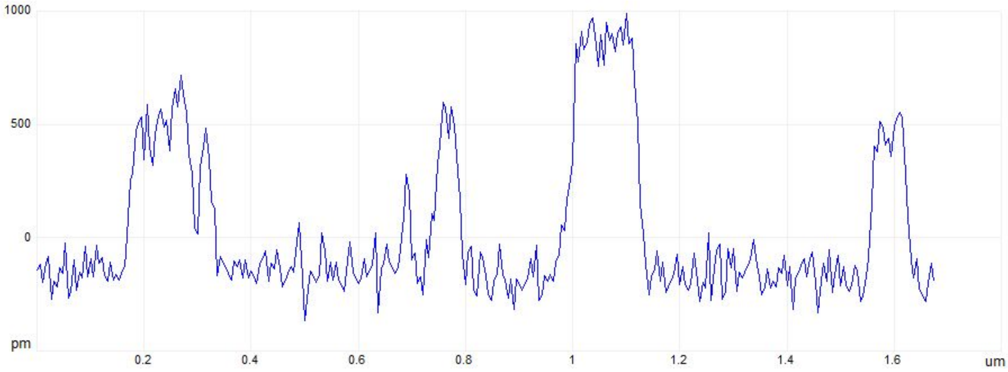


Height: around 0.7-1.3nm; Horizontal distance: 50-200nm

Europium Oxalate + 96% Et 10ml (1mg/ml) - *2hours sonication*

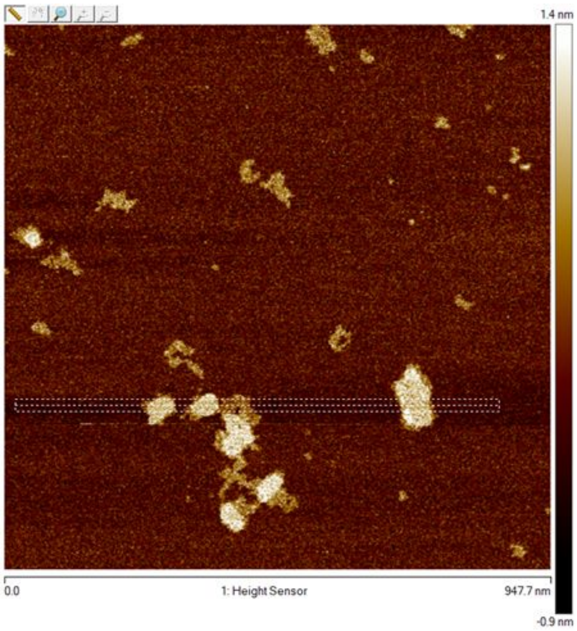


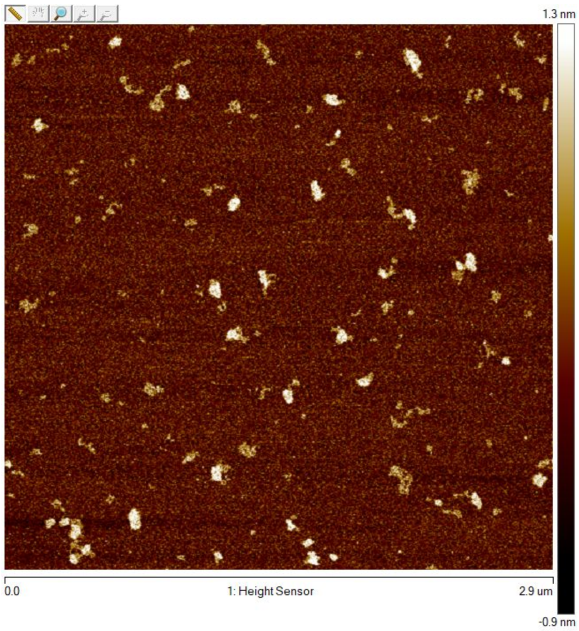

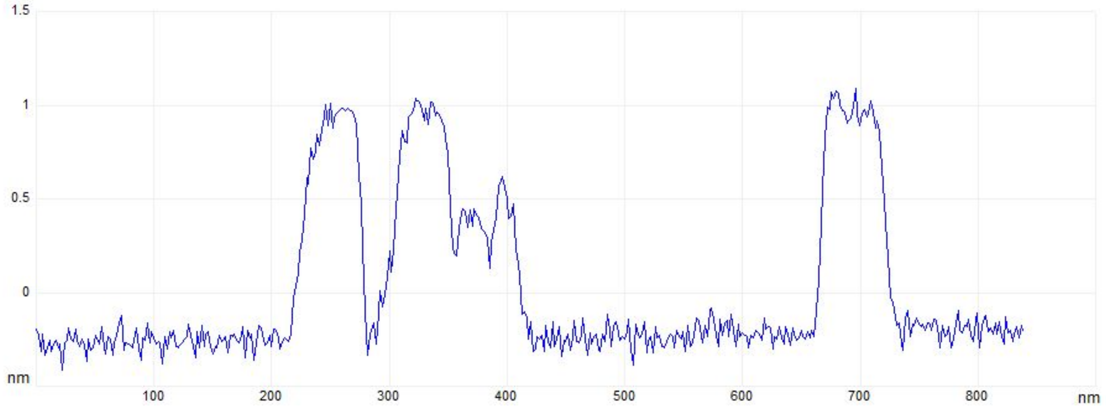


Height: 0,7-1,4 nm; Horizontal Distance: 30-130nm

Lutetium-Oxalate + 96% EtOH 10ml (1mg/ml) - *2hours sonication*

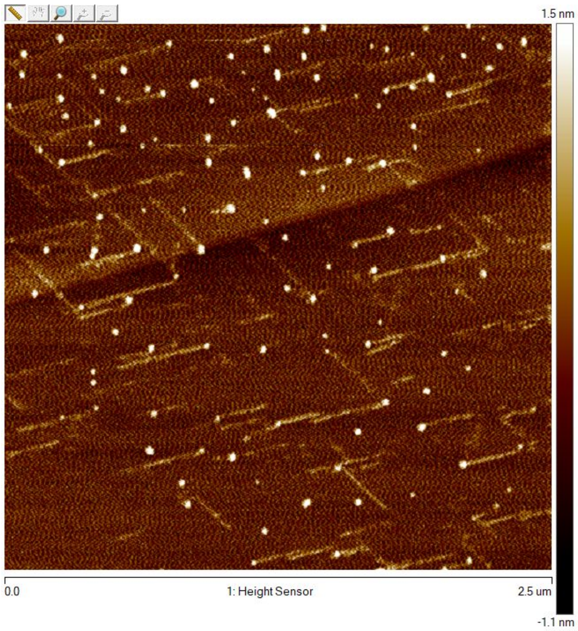


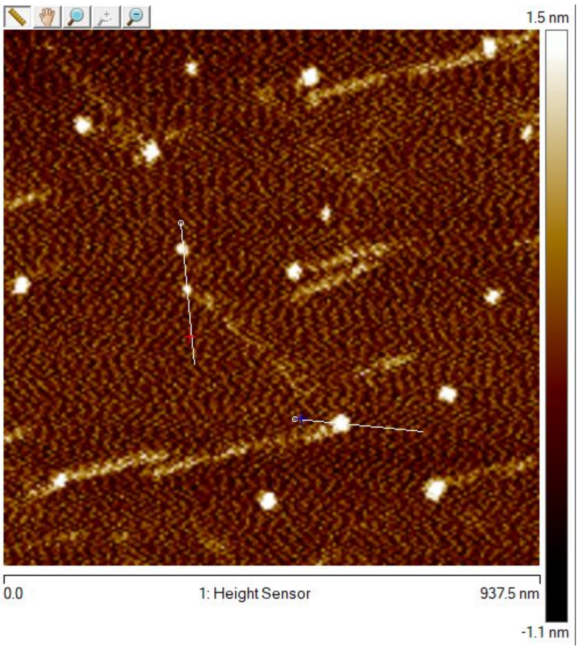


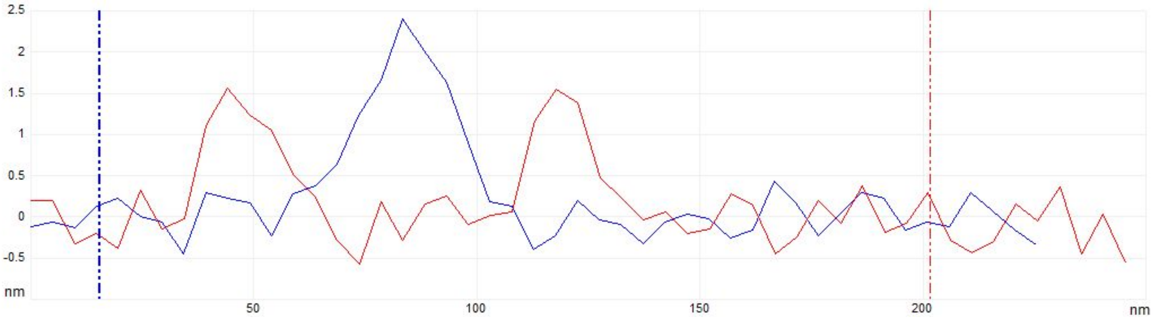


Height: 1.5-2.5nm; Horizontal distance: around 30nm

Ytterbium-Oxalate + 96% EtOH  10ml (1mg/ml) - *2hours sonication*

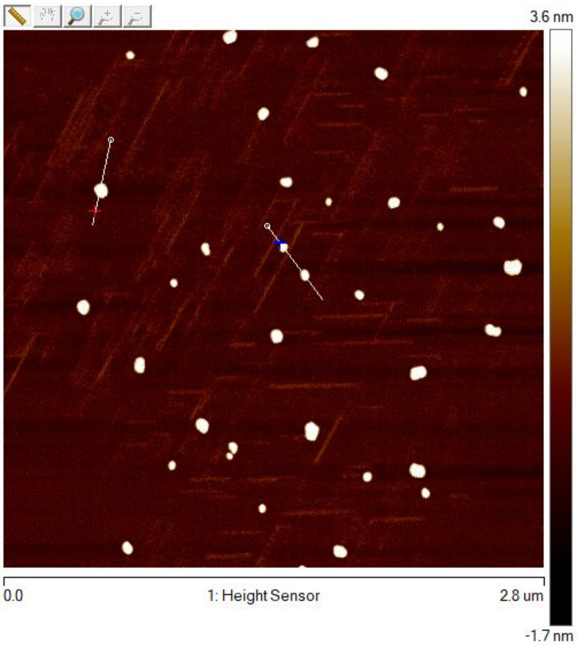


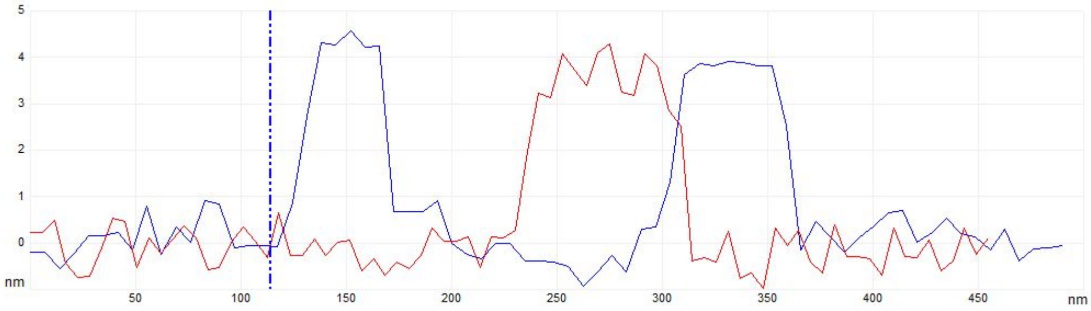


Height: 4-4.5nm; Horizontal distance: 50-100nm

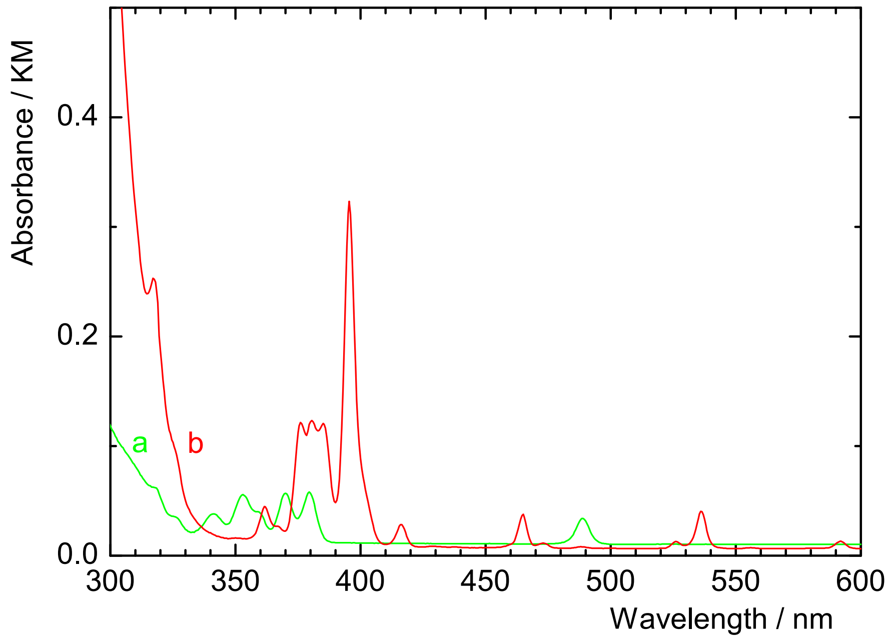


**Figure S5 Absorption spectra of Tb (a) and Eu (b) oxalate powders in the Kubelka-Munk-transformed reflectance scale.**

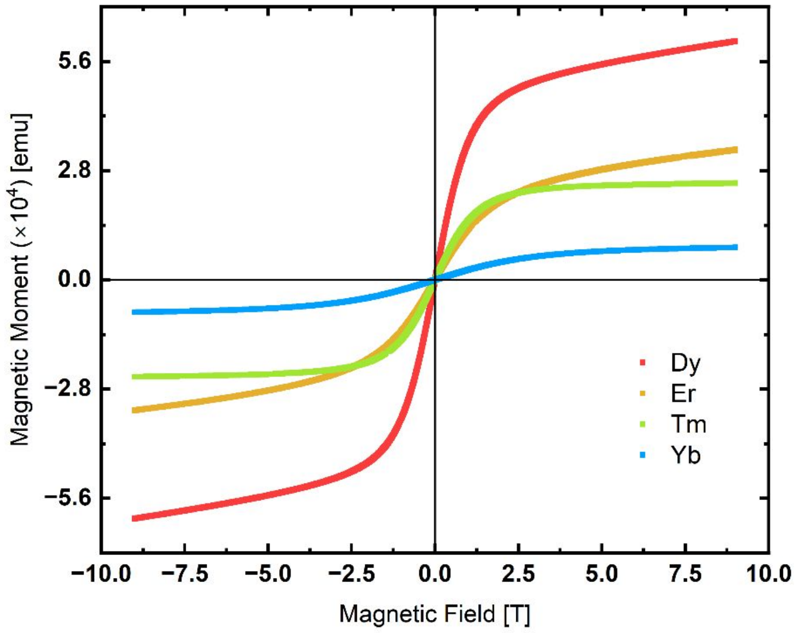


**Figure S6 Magnetisation vs. magnetic field measurement of Dy, Er, Tm and Yb oxalates at 4 K.**

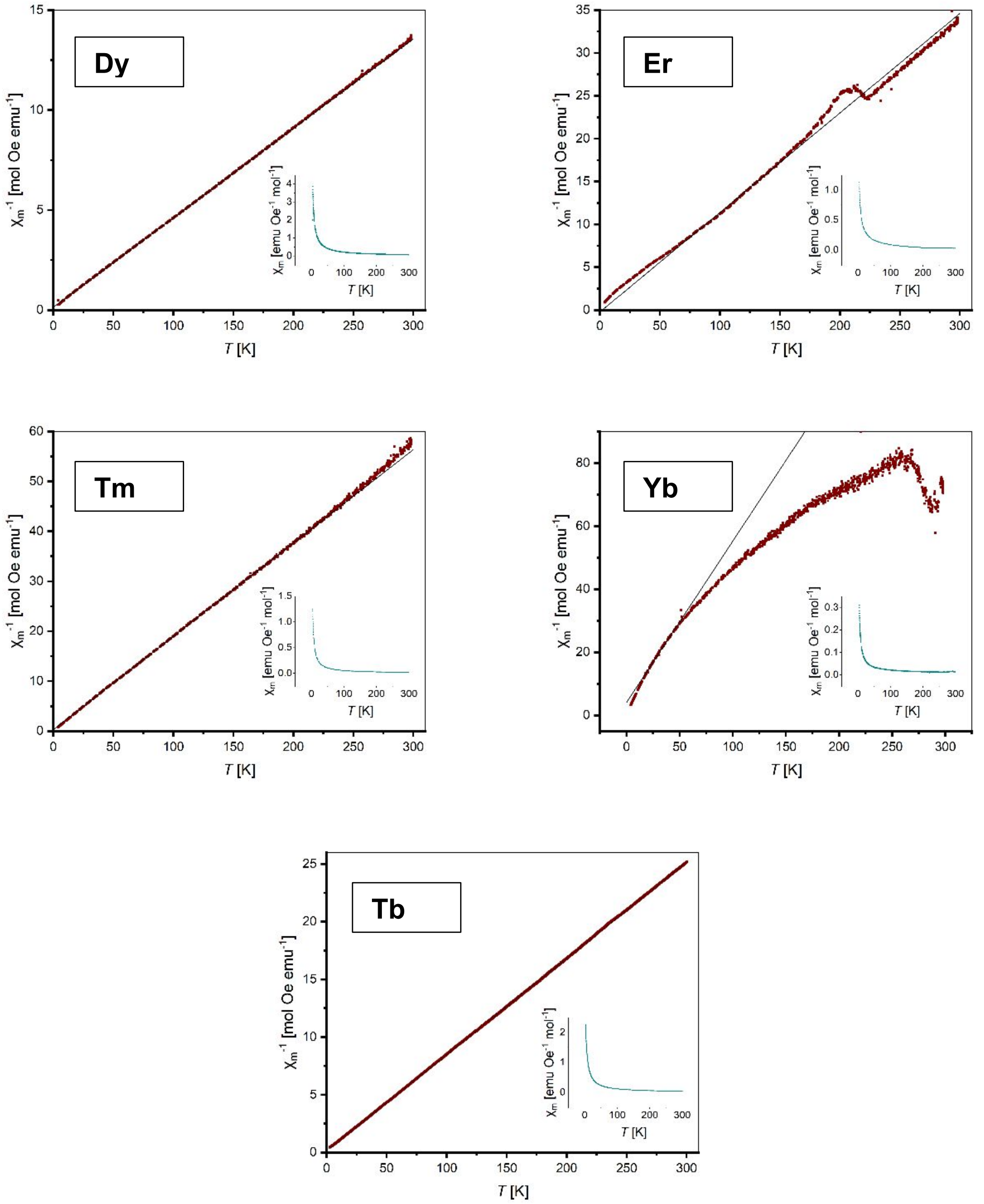


**Figure S7 Inverse molar magnetic susceptibility of Tb – Yb oxalates as a function of temperature, fitted by the Curie-Weiss law (red – experimental points, black line – CW fit, blue inset points – molar magnetic susceptibility).**

**Table S5 Measured effective magnetic moments of Tb to Yb oxalates compared to the theoretical values (and usual values) of the $Ln^{3+}$ free ions**

| $Ln^{III}$ | Theoretical effective moment [μB] | Measured effective moment [μB] |
|---|---|---|
| Tb | 9.72 (9.8) | 9.80 |
| Dy | 10.65 (10.6) | 13.36 |
| Er | 9.58 (9.5) | 8.57 |
| Tm | 7.56 (7.6) | 6.53 |
| Yb | 4.54 (4.5) | 3.95 |

**References:**


(1) E. Hansson, S. E. Sværen, J. Møller, G. Schroon the Rare Earth Carboxylatell, K. Leander, & C.-G. Swahn, Structural Studies on the Rare Earth Carboxylates. 16. The Crystal and Molecular Structure of Tetra-aquo Tris-oxalato Diytterbium(III) Dihydrate. *Acta Chemica Scandinavica*, **27** (1973) 823–834.

(2) E. Hansson & J. Albertsson, On the Crystal Structure of Neodymium Oxalate. *Acta Chemica Scandinavica*, **22** (1968) 1682–1683.

(3) E. Hansson, The Crystal and Molecular Structure of Neodymium(III) Oxalate 10,5-Hydrate. *Acta Chemica Scandinavica*, **24** (1970) 2969–2982.

(4) W. Ollendorff & F. Weigel, The crystal structure of some lanthanide oxalate decahydrates, Ln2(C2O4)3·10H2O, with Ln = La, Ce, Pr, and Nd. *Inorganic and Nuclear Chemistry Letters*, **5** (1969) 263–269. https://doi.org/10.1016/0020-1650(69)80196-0

(5) E. Hansson, The Crystal and Molecular Structure of Neodymium(III) Oxalate 10,5-Hydrate. *Acta Chemica Scandinavica*, **24** (1970) 2969–2982

(6) A. W. Wylie, Hydrated oxalates of some rare-earth elements. *Journal of the Chemical Society (Resumed)*, (1947) 1687–1692. https://doi.org/10.1039/jr9470001687.

(7) E. Löwenstein, Über Hydrate, deren Dampfspannung sich kontinuierlich mit der Zusammensetzung ändert. *Zeitschrift Fur Anorganische Chemie*, **63** (1909) 69–139. https://doi.org/https://doi.org/10.1002/zaac.19090630108.

(8) M. Camara, C. Daiguebonne, K. Boubekeur, T. Roisnel, Y. Gérault, C. Baux, F. Le Dret, & O. Guillou, Re-investigation of the Er3+–C2O42−–H2O system: from the classical ceramic precursor to a new nanoporous molecular material potential precursor. *Comptes Rendus Chimie*, **6** (2003) 405–415. https://doi.org/10.1016/S1631-0748(03)00057-2.

(9) I. Huskić, M. Arhangelskis, & T. Friščić, Solvent-free ageing reactions of rare earth element oxides: from geomimetic synthesis of new metal-organic materials towards a simple, environmentally friendly separation of scandium. *Green Chemistry*, **22** (2020) 4364. https://doi.org/10.1039/d0gc00454e.

(10) M. Ellart, F. Blanchard, M. Rivenet, & F. Abraham, Structural Variations of 2D and 3D Lanthanide Oxalate Frameworks Hydrothermally Synthesized in the Presence of Hydrazinium Ions. *Inorganic Chemistry*, **59** (2020) 491–504. https://doi.org/10.1021/ACS.INORGCHEM.9B02781/SUPPL_FILE/IC9B02781_SI_001.PD.

(11) S. F. Si & R. J. Wang, Poly[[tetraaquatri-μ-oxolato-dithulium] dihydrate]. *Acta Crystallographica Section E: Structure Reports Online*, **62** (2006) 1–7. https://doi.org/10.1107/S1600536805042376.

(12) A. E. Prozorovskii, A. B. Yaroslavtsev, & Z. N. Prozorovskaya, No Title. *Zh. Neorg. Khim*, **34** (1989) 2622.

(13)W. W. Wendlandt, Thermal Decomposition of Scandium, Yttrium, and Rare Earth Metal Oxalates. *Analytical Chemistry*, **30** (1958) 58–61. https://doi.org/10.1021/AC60133A015/ASSET/AC60133A015.FP.PNG_V03.

(14) S. Zhang, H. Ke, X. Liu, Q. Wei, G. Xie, & S. Chen, A nine-coordinated dysprosium(III) compound with an oxalate-bridged dysprosium(III) layer exhibiting two slow magnetic relaxation processes. *Chemical Communications*, **51** (2015) 15188–15191. https://doi.org/10.1039/C5CC05694B.

(15) Y. Zhang, I. Karatchevtseva, L. Kong, J. R. Price, N. Scales, S. Deen, & R. Zheng, Hydrothermal synthesis, structures and magnetic properties of two new holmium(III) oxalato complexes. *Journal of Coordination Chemistry*, **70** (2017) 2040–2051. https://doi.org/10.1080/00958972.2017.1320709/SUPPL_FILE/GCOO_A_1320709_SM8946.DOCX.

(16) I. V. Kalinina, A. L. Guschin, D. G. Samsonenko, A. V. Gerasimenko, M. N. Sokolov, & V. P. Fedin, Hexaaquatrioxalatodierbium(III) tetrahydrate: [{Er(H2O)3} 2(C2O4)3]·4H2O. *Acta Crystallographica Section E: Structure Reports Online*, **59** (2003). https://doi.org/10.1107/S1600536803018129.

(17) H. Steinfink & G. D. Brunton, The Crystal Structure of Erbium Oxalate Trihydrate. *Inorganic Chemistry*, **9** (1970) 2112–2115. https://doi.org/10.1021/IC50091A030/ASSET/IC50091A030.FP.PNG_V03
